# Integrating images, sounds, citizen science and AI to assess the biodiversity of the Atlantic Forest of Brazil

**DOI:** 10.1371/journal.pone.0360070

**Published:** 2026-10-07

**Authors:** Manoel Luis Costa, Claudia Mermelstein

**Affiliations:** 1 Instituto de Ciências Biomédicas, Universidade Federal do Rio de Janeiro, Rio de Janeiro, Rio de Janeiro, Brazil; 2 Colégio Brasileiro de Altos Estudos, Universidade Federal do Rio de Janeiro, Rio de Janeiro, Rio de Janeiro, Brazil; Instituto Federal de Educacao Ciencia e Tecnologia Goiano - Campus Urutai, BRAZIL

## Abstract

The Atlantic Forest is an endangered biome in Brazil with only 15% of its area remaining. Although it is reported that it has a high biodiversity and many endemic species, the biological composition of its territory remains largely unknown. Citizen science can speed up the description of the living organisms present in this biome and at the same time increase citizens’ perception of the importance of the conservation of this endangered tropical ecosystem. Here, we used citizen science resources to evaluate the biodiversity of an Atlantic Forest area in southeast Brazil. During a 6 year-period, we photographed plants, animals and fungi specimens using different approaches depending on the behavior and characteristics of each organism. We used easy-to-use mobile phones, night vision cameras, high-quality photographic cameras equipped with tele-macro lenses, and citizen science AI applications, such as iNaturalist and Merlin. We analyzed amphibians, arachnids, birds, fungi, insects, mammals, mollusks, plants, reptiles and crustaceans. We identified 492 species, some of which are threatened (4 plants, 4 mammals, 3 birds, and 1 reptile) according to the IUCN Red List categories of threatened species. We also identified some endemics and introduced species. Insects and plants were by far the groups with the highest numbers of species registered, followed by birds, fungi and arachnids. Birds were the group with the highest numbers of observations, followed by insects and plants. Amphibians were the group with more endemic species, with 60% of their species endemic in Brazil. Lepidoptera (butterflies and moths) correspond to 50% of the insect species found in the area. The collection of our data demonstrates that it is easily feasible to use a technological approach to register and identify the species found in small properties and we provide an easy guide for beginners. To our knowledge this is the first description of the biodiversity across several taxonomic categories of the Brazilian Atlantic Forest using an integrated approach combining digital observations, AI-assisted species identification, and citizen-science community validation. Our data could have an impact on the engagement of citizens on the management and conservation of Atlantic Forest sustainability in Brazil and in other parts of the globe.

## Introduction

Citizen Science (CS) is the public participation in the gathering of scientific knowledge [1,2]. On one hand, it should be viewed as a way to foster scientific education and public commitment to science, but it is also about people, without formal professional training, collecting scientific relevant data [3]. Our focus on this study is on the quality of biodiversity data that can be gathered by CS. The type and amount of information generated with CS can vary within areas of science, such as in ecology, geology and astronomy. One of the reasons for the recent spread of CS is the advance of technology, both hardware (cameras and powerful cellphones) and software (Artificial Intelligence to allow species recognition). Both price reduction and ease of use of these technologies have allowed CS individual and community initiatives, empowering a larger public to produce scientific information. It is reasonable to expect that CS will reach beyond the limits of traditional science, allowing data to be produced by a much larger public. Furthermore, scientists not trained in a given field can participate in data collection in other areas and therefore CS can also be considered as a transdisciplinary initiative [4].

One promising area where CS can contribute is in the assessment of biodiversity. The use of photography and CS can accelerate biodiversity studies [5]. Biodiversity reduction and habitat loss are some of the main problems of the world at the moment. One of the most affected environments in the world is the Atlantic Forest in Brazil. The Atlantic Forest (“Mata Atlântica”) is a tropical biome that spans most of the Brazilian coast. In southeast Brazil it occurs in the mountain regions, with a high level of rain and high number of endemic species. Remarkably, in Brazil, only ~15% of the original Atlantic Forest area remains [6]. Furthermore, there is an extensive reduction in the number of species, and several species are threatened with extinction [7]. This disturbing scenario drove us to study the biodiversity of this endangered biome with a hands-on approach, taking advantage of combined recent technological innovations. We decided to evaluate, in a CS perspective, the diversity of species of a privately owned property, an Atlantic Forest area in the mountains of Petropolis, near the city of Rio de Janeiro (Brazil).

There are several ways to assess the biological species in a given area: photographs, physical traps, visual observation, and sound records. Among these techniques, photographs have the advantage of being completely harmless and non-invasive to animals. The modern photographic capabilities allow for night images, using infrared light, and long-distance images, based on stabilized telephoto lenses. Moreover, photographs are now digital and with high resolution. The change from negative to digital imaging revolutionized photography, particularly in terms of cost and quality. Nowadays, the cost of obtaining and storing digital images is very low, and the use of images has pervaded our culture. Animal registration began very early (circa 1884) after the invention of photography, but to catch images of wild animals in nature has always been a challenge [8,9]. This burden has been lessened by digital photography and the reduction in cost of high-quality telephoto lenses, which are nowadays of very high light sensibility and digitally stabilized. Common cellphones now have powerful high-resolution cameras and large memory storage capabilities. In particular, camera traps with autonomous register capability were developed, and nowadays have very short response times (in the range of 0.5 seconds), infrared nocturnal detection and imaging and low cost.

Several animals use sound to communicate, which allows dedicated software, such as Merlin, to identify species with high accuracy using simple cell phones. Sound recording is also non disturbing, provided that the user does not playback the recorded sound.

While the ability to register images and sounds of natural life has increased recently, the non-professional user still needs to be able to identify the species that were recorded. The recent development of Artificial Intelligence (AI) led to the development of software that can identify species with high precision. In particular, the recognition uses local data based on geographic position to improve the reliability of the species identification.

Among the various species identification applications, we choose iNaturalist, Merlin and Addax. The iNaturalist (iNat) platform is a successful example of the power that CS can have in the acceleration of biodiversity research [10]. iNat is a nonprofit social network of naturalists and citizen scientists built on the concept of mapping and sharing observations of biodiversity, developed by the National Geographic Society together with the California Academy of Sciences. The importance of gathering biodiversity information around the world is enormous during the global warming and extreme climate changes that our planet has been undergoing in the last decades [11]. Biodiversity data, such as those generated through CS initiatives, are playing an increasingly important role in providing the necessary data for biodiversity assessments [12,13]. iNat is contributing to the increase in the collection of biodiversity data everywhere by common citizens [14]. Merlin is an automated bird-sound classifier that runs on mobile devices. It has been developed by the Cornell Lab of Ornithology. Merlin identifies birds in a region by combining the specific GPS location and date with the vast eBird database of bird observations, using this data to filter possibilities by size, color, and time of year for its powerful Sound ID (using machine learning for sounds). We also used the software Addax AI to classify the assembly of our data [15]. Addax has been used to process camera trap data in several protected areas worldwide. Interestingly, both Merlin and Addax use directly the raw images registered by the hardware, while iNat uses images processed by the user (images are usually cropped and their brightness and contrast corrected). All the applications are free or on a voluntary contribution basis.

Scientific articles about biodiversity in a given region tend to be restricted to a particular group, and therefore usually based on a particular methodology, such as camera traps. One of the most important achievements of our study was the combined approach of the use of mobile phone cameras, high resolution photographic cameras, night vision cameras and AI-applications for species recognition, for the assessment of the biodiversity of an endangered area of the Atlantic Forest biome. We assume that these resources are within reach of non-professional naturalists, and to stimulate similar initiatives, here we provide an easy guide to the assessment of biodiversity in the Atlantic Forest of Brazil.

We collected data from Plantae, Animalia and Fungi, in a 6 year-survey on the biodiversity of the Brazilian Atlantic Forest. We analyzed amphibians, arachnids, birds, insects, mammals, mollusks, reptiles and crustaceans. Due to their intrinsic characteristics, we used complementary approaches to observe and acquire information about organisms from distinct groups. Plants were photographed mostly with cellphones; birds were registered with professional cameras with tele-objective lenses and with the cellphone app Merlin (https://merlin.allaboutbirds.org/). Mammals were mainly registered with automated camera traps, either during day or night. Small animals (insects, arachnids and crustacea) and fungi were photographed with professional cameras with macro lenses or with cellphones. All specimens were afterwards deposited and classified in the iNaturalist application (https://www.inaturalist.org/) or classified in the Merlin application. Images from camera traps were also analyzed with Addax software.

## Materials and methods

### Study area

We produced data on the biodiversity of an Atlantic Forest region located in southeast Brazil (22°15’46.4“S 43°01’43.8”W, Brejal, Petrópolis, Rio de Janeiro, RJ, Brazil) (Fig 1A-C). This property has been owned by the authors since 1960.

**Fig 1:**
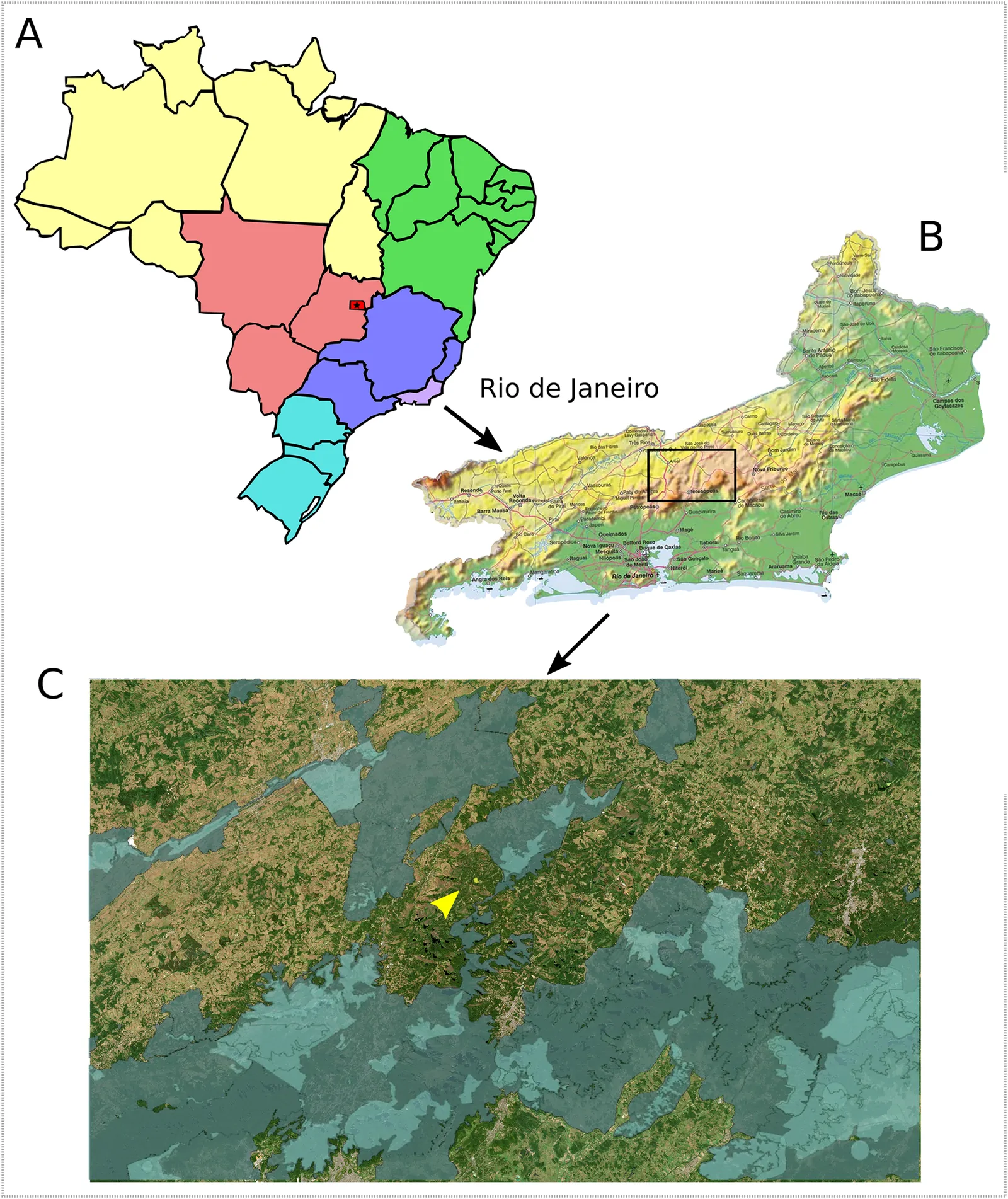
Map of the study area. Map showing the region studied. In A, the states of Brazil are represented. In B, the geography of the state of Rio de Janeiro is shown, with the inset showing the area of the present studied in more detail in C. The image in C shows a satellite view of the area, with protected areas shown in blue (light blue indicates that the area has multiple protection conditions). The study area is shown in white, pointed by the yellow arrowhead. Credits: 1A: https://publicdomainvectors.org/pt/vetorial-gratis/Mapa-do-Brasil-com-a-imagem-vetorial-de-lenda/11574.html. Acessed in Aug 2026. 1B: Source: IBGE. Base cartográfica contínua do Brasil ao milionésimo, escala 1:100 000 – BCIM. Versão 2014. Rio de Janeiro, 2014. Avaliable at: https://atlasescolar.ibge.gov.br/unidades-da-federacao/21715-rio-de-janeiro-rj.html. Accessed in Aug 2026). 1C: Source: Sentinel 2 L2a at Copernicus Sentinel Data and Service Information. Contains modified Copernicus Sentinel data 2026. Available at: link.dataspace.copernicus.eu/e017. Accessed in Aug 2026).

The Atlantic Forest is rich in biodiversity, hosting numerous endemic species. The selected area is located at an altitude of 910 meters, in the biogeographical sub-region “Serra do Mar” [16]. Its vegetation, part of dense broad leaf forest, includes remnants of the large original forest, regions with restored forest, pasture areas and vegetable production patches. The Portuguese name “Brejal” means marsh, since the area has many waterlogged soils with nutrient-rich organic matter. Brejal has several small water streams. This area has a Tropical Highland Climate (“Clima de Altitude Tropical”) and has two very distinct seasons: summer (December-March) and winter (June-September). Summer days are quite hot (26–30°C) and humid, and summer nights are characterized by heavy rainstorms. Conversely, winter days and nights are cold (3–15°C) and less humid, with very sparse rainfall. The area is basically occupied by small houses and farms, with a familiar agriculture system producing vegetables, with focus on organic farming. The area is relatively sparsely populated, but this has been changing in the last three decades. There are several protected areas nearby: the Parque Municipal Montanhas de Teresópolis and the Área de Proteção Ambiental Maravilha (marked in blue in Fig 1C). These areas, together with the large Parque Nacional da Serra dos Órgãos, the Reserva Municipal do Tinguá, and other protected areas, comprise the Mosaico Central Fluminense (a government initiative to bring together several protected areas).

### Image and sound acquisition

Below we describe the four types of photographic devices we used in this study to capture images of living organisms.

1 – Reflex camera: Photographic camera Nikon D3200 DSLR (Japan) equipped with a 150–600 mm lens (Sigma, Japan) and a 70–300 mm AF Tele-Macro lens (Tamron, Japan). The photographs have a resolution of 24 Mpixels.

2 – Surveillance camera: WIFI Camera Outdoor 360° PTZ Speed Wireless IP Camera CCTV 4X Digital Zoom Audio Network Surveillance CAM. Images have a resolution of 2.1 to 3.7 Mpixels. Up to 4 surveillance cameras were used.

3 – Camera trap: Suntek mini 301 camera trail wildlife outdoor night vision photo color, with a resolution of 20 Mpixels. Up to 4 camera traps were used.

4 – Camera from an Android (version 14) mobile phone Motorola Edge 30 neo, with a resolution of 16 Mpixels. The camera system has a sensor with Optical Image Stabilization (OIS) with an ultrawide lens that also functions as a macro camera. The same cellphone was used to record bird audios.

We used different approaches to observe, take pictures, make movies and listen to sounds of different living organisms (Table 1). All images and sounds were registered solely by the authors. Birds were usually registered with long-focal-length lenses in reflex cameras and Merlin App (https://merlin.allaboutbirds.org/) in a cell phone. Mammals were usually registered with camera traps. All animals were registered without bait. Reptiles were registered with reflex cameras and with camera traps. Amphibians, arachnids, insects and plants were registered with cell phones and reflex cameras. Mollusca were registered with a cell phone. Crustacea were registered with reflex cameras. Fungi were registered with cell phone cameras, pictures were taken of the top, lateral and bottom of all fungi to facilitate their classification. All images were identified in the iNat application.

**Table 1:** List of the photographic devices used in this study and their applications.

Live organism group	Photographic Device and Identification Application
Birds	long-focal-length camera lens, iNat, Merlin and Addax
Mammals	long-focal-length camera lens, night camera trap, iNat and Addax
Reptiles	long-focal-length camera lens and iNat
Amphibians	cell phone, long-focal-length camera lens and iNat
Arachnids	cell phone, long-focal-length camera lens and iNat
Mollusca	cell phone and iNat
Fungi	cell phone and iNat
Insects	cell phone and iNat
Plants	cell phone, long-focal-length camera lens and iNat

The workflow began with photographs and sound recording, followed by appropriate software processing in the case of sounds and camera traps, followed by the upload of photographs and sounds to the iNaturalist platform. In the iNat site, recordings were initially classified by automated AI, and afterwards classified in the species level by identifiers, in order to achieve Research Grade (Fig 2).

**Fig 2:**
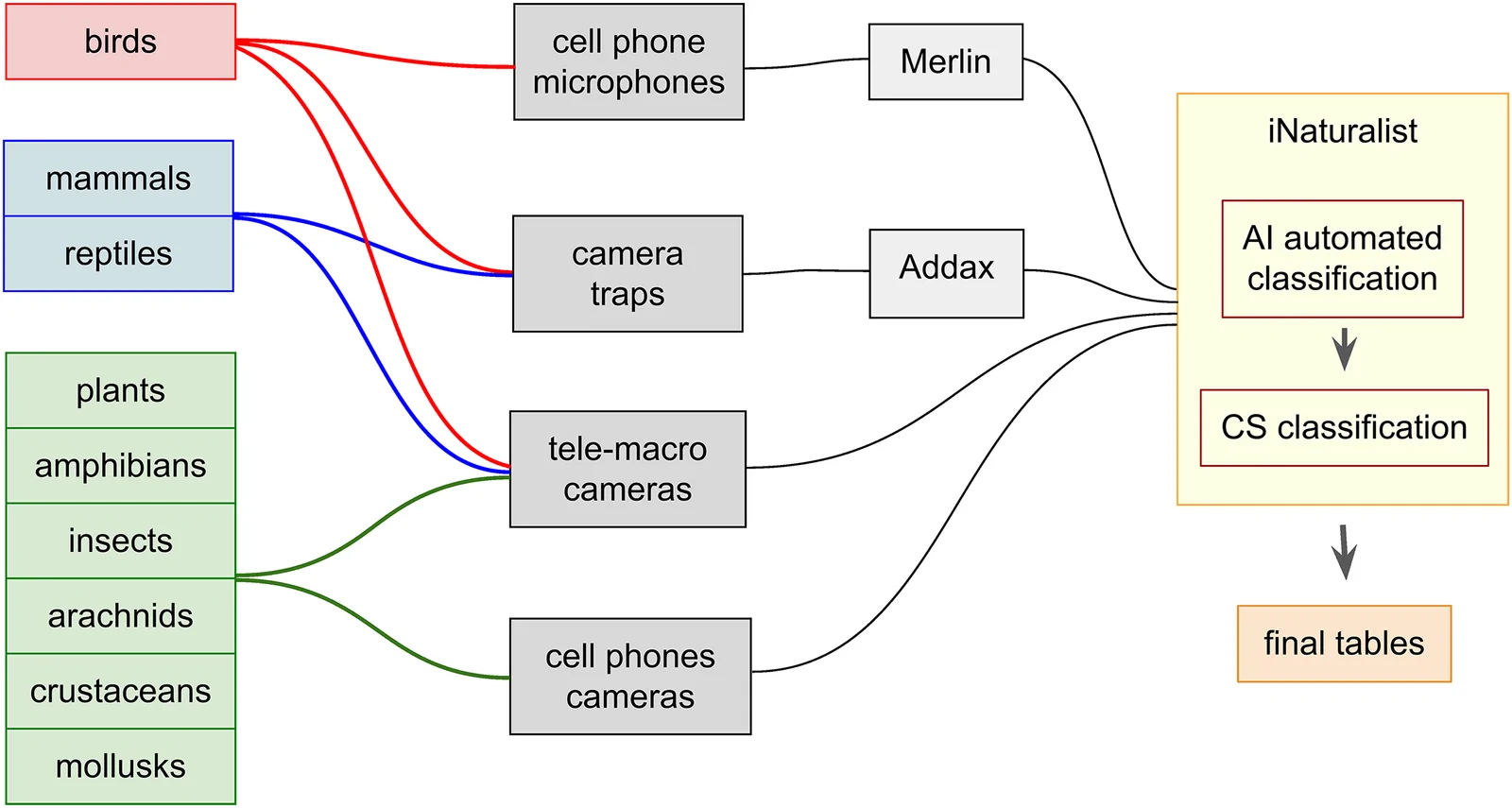
Images and sound recording and analysis workflow. The workflow began with photographs and sound recording, followed by appropriate software processing in the case of sound and camera traps, followed by the upload to iNaturalist site. In the iNaturalist site, recordings were initially classified by automated AI, and afterwards classified by identifiers, in order to achieve Research Grade.

To stabilize the camera for bird pictures, we used a monopod in the reflex camera coupled with the tele-macro lens. To store the captured images, SD memory cards with 64 GBytes were used in the reflex camera and in the camera traps (devices #1, #2 and #3, above described). Birds were usually registered very early in the morning (between 5 and 8 am) and at the end of the afternoon (between 4 and 6 pm), which are the peaks of birds’ daily activities in this area.

The night vision camera traps were set on 10 different locations in the studied area (approximately 200 meters apart from each other) and always near the ground to favor the capture of terrestrial animal images. A total area of 100,000 square meters was covered in this study. Considering the number of camera traps and the time they were active, we estimate that we accumulated around 1,600 camera trap days, which produced approximately 10,000 images. Around 4,000 of these were empty images, and around 3,800 were wild animal observations (around 2,200 were humans or domestic animals). Surveillance cameras register videos, out of which we selected animal images for iNat. We estimate that we accumulated around 2,200 surveillance camera days.

### Data assembly

Images were regularly transferred from the memory cards to a computer where they were stored in an external high-capacity hard drive. Some images were cropped and adjusted for brightness and contrast (particularly with gamma correction) using the public domain software ImageJ (https://imagej.net/ij/). Images were manually selected for upload to the iNaturalist application (https://www.inaturalist.org/) with the information of the date of the photograph and the geographic coordinates. They were then classified using the iNat algorithms to match the best identification of the images to the species level. Sometimes, iNat could not provide a species-level identification. Afterwards, the observations were either (i) classified by other iNat users conferring the image with “research-grade” record, (ii) left unclassified, or (iii) were subjected to changes in the classification made by other iNat users. These suggested changes were then analyzed by the authors of this study and accepted or not. In most cases we accepted the suggestions, which were usually made by specialists, scientists, naturalists or people with high experience in specific species. We acknowledge that the identification of species in iNat is not perfect and errors exist, but the majority of identifications are made by taxonomic experts [17]. More than 90% of the observations used in this study were identified at the species level, with the exception of some living groups (arachnids and fungi) that had a lower percentage of classification in iNat. We downloaded all 1,515 iNat records from the “Brejal-Petrópolis-RJ and its Biodiversity” Project on December 27, 2025, corresponding to a 6 year-survey (2020–2025), and these data can be found in S1 File. From this spreadsheet we manually prepared tables listing species for amphibians, arachnids, birds, crustaceans, insects, mammals, mollusks and reptiles. We included in each table the information related to “species name” and “popular name”. For some tables, we also included the information “introduced in the region”, “endemic”, and “threatened”. In the case of Birds, we also included information on species identified by the Merlin app. We used the data from the iNat spreadsheet to calculate the relative frequency of each species. It should be noted that new observations are still being uploaded to iNat, which will continue to be freely available (user: claudia_mermelstein), at https://www.inaturalist.org/observations?project_id=brejal-petropolis-rj-e-sua-biodiversidade&user_id=claudia_mermelstein. For the analysis of relative frequency of automatic observations with camera traps, we used AddaxAI software (https://addaxdatascience.com/addaxai/) to classify all image files. Another reason for the use of automated image classification with Addax, in the case of camera traps, is that several images are captured without any animals. These empty images can occupy a lot of unnecessary space. Addax automated image analysis identified images without animals and classified the remaining image files in distinct species. After the automated classification, images were manually checked to confirm accuracy.

## Results and discussion

Here we analyzed the biodiversity of a region located in the Atlantic Forest of southeast Brazil (Fig 1) during a 6-year period of observations (from August 2020 to January 2026). The combination of a photographic camera equipped with telephoto lenses, surveillance cameras for outdoors, camera traps with night vision, and mobile phones with cameras, with citizen science and Artificial Intelligence software (iNaturalist, Merlin and Addax), allowed us to register, identify and classify 492 species. We photographed plants (146), fungi (33) and animals (313) specimens (Figs 3–5 and Tables 2–9). Amongst animals, we analyzed the invertebrates Arachnida (19), Crustacea (1), Insects (146), and Mollusca (3); and the vertebrates Amphibians (9), Birds (100), Mammals (20), and Reptilia (15). In total, we analyzed 10 groups of living organisms, which are illustrated in Fig 3. iNat application was essential for the identification of these species from photographic images, with 495 iNat identifiers (individual users who help to identify the species) involved in the process. Some bird species, which were difficult to photograph, were identified by their sound with Merlin. Images from camera traps were also analyzed and classified with Addax software.

**Table 2:** List of all bird species identified in this study in the Atlantic Forest of southeast Brazil.

	Species name	Popular name (in Portuguese)	Popular name (in English)	iNat	Merlin
1	*Anabazenops fuscus*	Trepador-Coleira	White-collared Foliage-gleaner	X	
2	*Aramides saracura*	Saracura-Do-Mato	Slaty-breasted wood rail	X	X
3	*Astur bicolor*	Gavião-Bombachinha- Grande	Bicolored Hawk	X	
4	*Baryphthengus ruficapillus*	Juruva	Rufous-capped Motmot	X	X
5	*Basileuterus culicivorus*	Pula-Pula	Golden-crowned Warbler	X	X
6	*Batara cinerea*	Matracão	Giant Antshrike	X	X
7	*Campephilus robustus*	Pica-Pau-Rei	Robust Woodpecker	X	
8	*Camptostoma obsoletum*	Risadinha	Southern Beardless-Tyrannulet	X	X
9	*Cathartes aura*	Urubu-de-Cabeça- Vermelha	Turkey Vulture	X	
10	*Celeus flavescens*	Pica-Pau-de-Cabeça- Amarela	Blond-crested Woodpecker	X	
11	*Chiroxiphia caudata*	Tangará	Swallow-tailed Manakin	X	X
12	*Colaptes melanochloros*	Pica-Pau-Verde- Barrado	Green-Barred Woodpecker	X	
13	*Colonia colonus*	Viuvinha	Long-tailed Tyrant	X	
14	*Conopophaga lineata*	Chupa-Dente	Rufous Gnateater	X	X
15	*Contopus cinereus*	Papa-Moscas-Cinzento	Southern Tropical Pewee	X	X
16	*Crypturellus obsoletus*	Inhambuguaçu	Brown Tinamou	X	X
17	*Cyanocorax cristatellus*	Gralha-Do-Campo	Curl-crested Jay	X	
18	*Cyanoloxia brissonii*	Azulão	Ultramarine Grosbeak	X	
19	*Cyclarhis gujanensis*	Pitiguari	Rufous-browed Peppershrike	X	X
20	*Dacnis cayana*	Saí-Azul	Blue Dacnis	X	
21	*Daptrius chimachima*	Carrapateiro	Yellow-headed Caracara	X	
22	*Dendrocolaptes platyrostris*	Arapaçu-Grande	Planalto Woodcreeper	X	
23	*Dendroma rufa*	Limpa-Folha-de-Testa- Baia	Buff-fronted foliage-gleaner	X	X
24	*Drymophila ochropyga*	Choquinha-de-Dorso- Vermelho	Ochre-rumped antbird		X
25	*Dryocopus lineatus*	Pica-Pau-de-Banda- Branca	Lineated woodpecker	X	X
26	*Dysithamnus mentalis*	Choquinha-Lisa	Plain antvireo	X	X
27	*Empidonomus varius*	Peitica	Variegated flycatcher	X	
28	*Geranoaetus albicaudatus*	Gavião-de-Rabo-Branco	White-tailed hawk	X	
29	*Geranospiza caerulescens*	Gavião-Pernilongo	Crane hawk	X	
30	*Glaucidium brasilianum*	Caburé	Ferruginous pygmy owl	X	
31	*Hemithraupis ruficapilla*	Saíra-Ferrugem	Rufous-headed tanager	X	
32	*Hemitriccus diops*	Olho-Falso	Drab-breasted bamboo tyrant	X	X
33	*Herpetotheres cachinnans*	Acauã	Laughing falcon	X	
34	*Hylophilus poicilotis*	Verdinho-Coroado	Rufous-crowned greenlet	X	X
35	*Ictinia plumbea*	Sovi	Plumbeous kite	X	
36	*Ilicura militaris*	Tangarazinho	Pin-tailed manakin	X	
37	*Lathrotriccus euleri*	Enferrujado	Euler’s Flycatcher	X	X
38	*Lepidocolaptes squamatus*	Arapaçu-Escamoso	Scaled woodcreeper	X	
39	*Leptodon cayanensis*	Gavião-Gato	Gray-headed kite	X	
40	*Leptopogon amaurocephalus*	Cabeçudo	Sepia-capped flycatcher	X	
41	*Leptotila verreauxi*	Juriti-Pupu	White-tipped dove	X	X
42	*Lochmias nematura*	João-Porca	Sharp-tailed streamcreeper	X	X
43	*Lurocalis semitorquatus*	Tuju	Short-tailed nighthawk		X
44	*Mackenziaena severa*	Borralhara	Tufted antshrike	X	X
45	*Malacoptila striata*	Barbudo-Rajado	Crescent-chested puffbird	X	
46	*Manacus manacus*	Rendeira	White-bearded manakin	X	
47	*Megarynchus pitangua*	Neinei	Boat-billed flycatcher	X	X
48	*Mionectes rufiventris*	Abre-Asa-de-Cabeça-Cinza	Grey-hooded flycatcher	X	
49	*Myiodynastes maculatus*	Bem-Te-Vi-Rajado	Streaked flycatcher	X	X
50	*Myiozetetes similis*	Bentevizinho-de- Penacho-Vermelho	Social flycatcher		X
51	*Pachyramphus castaneus*	Caneleiro	Chestnut-crowned becard	X	
52	*Pachyramphus validus*	Caneleiro-de- Chapéu-Preto	Crested becard	X	
53	*Patagioenas picazuro*	Pomba-Asa-Branca	Picazuro pigeon	X	X
54	*Penelope obscura*	Jacuguaçu	Dusky-legged guan	X	X
55	*Phaethornis eurynome*	Rabo-Branco-de- Garganta-Rajada	Scale-throated hermit	X	
56	*Piaya cayana*	Alma-de-Gato	Squirrel cuckoo	X	X
57	*Piculus aurulentus*	Pica-Pau-Dourado	Yellow-browed woodpecker	X	
58	*Pionus maximiliani*	Maitaca-Verde	Scaly-headed parrot	X	X
59	*Pitangus sulphuratus*	Bem-Te-Vi	Great Kiskadee	X	X
60	*Platyrinchus mystaceus*	Patinho	White-throated spadebill	X	
61	*Poecilotriccus plumbeiceps*	Tororó	Ochre-faced tody-flycatcher	X	
62	*Primolius maracana*	Maracanã	Blue-winged macaw	X	
63	*Procnias nudicollis*	Araponga	Bare-throated bellbird		X
64	*Psarocolius decumanus*	Japu	Crested oropendola	X	X
65	*Psittacara leucophthalmus*	Periquitão	White-eyed parakeet	X	X
66	*Pteroglossus bailloni*	Araçari-Banana	Saffron toucanet	X	
67	*Pygochelidon cyanoleuca*	Andorinha-Pequena- de-Casa	Blue-and-white swallow	X	X
68	*Pyriglena leucoptera*	Papa-Taoca-Do-Sul	White-shouldered fire-eye	X	X
69	*Ramphastos toco*	Tucanuçu	Toco Toucan	X	X
70	*Rupornis magnirostris*	Gavião-Carijó	Roadside Hawk	X	X
71	*Saltator maximus*	Tempera-Viola	Buff-throated saltator		X
72	*Saltator similis*	Trinca-Ferro	Green-winged saltator		X
73	*Sclerurus scansor*	Vira-Folha-Cearense	Rufous-breasted leaftosser		X
74	*Sittasomus griseicapillus*	Arapaçu-Verde	Olivaceous woodcreeper	X	X
75	*Spizaetus tyrannus ssp. tyrannus*	Gavião-Pega- Macaco	Black hawk-eagle	X	X
76	*Stilpnia cayana*	Saíra-Amarela	Burnished-buff tanager	X	
77	*Strix virgata*	Coruja-do-Mato- neotropical	Mottled owl		X
78	*Synallaxis cinerascens*	Pi-Puí	Grey-bellied spinetail		X
79	*Synallaxis ruficapilla*	Pichororé	Rufous-capped spinetail	X	
80	*Syndactyla rufosuperciliata*	Trepador-Quiete	Buff-browed foliage-gleaner		X
81	*Tachyphonus coronatus*	Tiê-Preto	Ruby-crowned tanager	X	X
82	*Tangara cyanoventris*	Saíra-Douradinha	Gilt-edged tanager	X	
83	*Tangara desmaresti*	Saíra-Lagarta	Brassy-breasted tanager	X	
84	*Thalurania glaucopis*	Beija-Flor-de- Fronte-Violeta	Violet-capped woodnymph	X	
85	*Thamnophilus caerulescens*	Choca-da-Mata	Variable antshrike	X	X
86	*Thraupis ornata*	Sanhaço-de- Encontro-Amarelo	Golden-chevroned tanager	X	
87	*Thraupis sayaca*	Sanhaço-Cinzento	Sayaca tanager		X
88	*Tolmomyias sulphurescens*	Bico-Chato-de- Orelha-Preta	Yellow-olive flatbill	X	
89	*Trichothraupis melanops*	Tiê-de-Topete	Black-goggled tanager	X	
90	*Troglodytes musculus*	Corruíra	Southern house wren	X	X
91	*Trogon surrucura*	Surucuá-Variado	Surucua trogon	X	X
92	*Trogon viridis*	Surucuá-de-Barriga- Amarela	Green-backed trogon	X	
93	*Turdus albicollis*	Sabiá-Coleira	White-necked thrush	X	X
94	*Turdus leucomelas*	Sabiá-Barranco	Pale-breasted thrush	X	X
95	*Turdus rufiventris*	Sabiá-Laranjeira	Rufous-bellied thrush	X	X
96	*Tyranus melancholicus*	Suiriri	Tropical kingbird		X
97	*Veniliornis maculifrons*	Pica-Pau-de-Testa- Pintada	Yellow-eared woodpecker	X	
98	*Xenops rutilans*	Bico-Virado-Carijó	Streaked xenops	X	X
99	*Xiphorhynchus fuscus*	Arapaçu-Rajado	Lesser woodcreeper	X	X
100	*Zonotrichia capensis*	Tico-Tico	Rufous-collared sparrow		X

**Table 3:** List of all fungi species identified in this study in the Atlantic Forest of southeast Brazil.

	Species name
1	*Armillaria puiggarii*
2	*Auricularia fuscosuccinea*
3	*Cerrena zonata*
4	*Cladonia confusa*
5	*Clitocybula azurea*
6	*Cotylidia aurantiaca*
7	*Crepidotus applanatus*
8	*Earliella scabrosa*
9	*Favolus brasiliensis*
10	*Flabellophora obovata*
11	*Flavopunctelia flaventior*
12	*Ganoderma australe*
13	*Ganoderma lobatum*
14	*Ganoderma resinaceum*
14	*Geastrum saccatum*
15	*Gymnopilus liquiritiae*
16	*Gymnopilus luteofolius*
17	*Gymnopilus penetrans*
20	*Gymnopilus sapineus*
21	*Gymnopus foetidus*
22	*Herpothallon rubrocinctum*
23	*Irpex rosettiformis*
25	*Laetiporus gilbertsonii*
27	*Leucocoprinus fragilissimus*
28	*Leucocoprinus ianthinus*
29	*Oudemansiella cubensis*
30	*Phallus indusiatus*
31	*Pseudoclitocybe cyathiformis*
32	*Trametes hirsuta*
33	*Trametes sanguinea*

**Table 4:** List of all arachnid species identified in this study in the Atlantic Forest of southeast Brazil.

	Species name	Popular name Portuguese	Popular name English
1	*Araneus horizonte*	Aranha-Orbiculária	Orbicularis-Occiput-Spider
2	*Argiope argentata*	Aranha-de-Prata	Silver-Argiope
3	*Eriophora edax*	Aranha-Tecedeira	Orb-Weaver
4	Family *Lycosidae*	Aranha-Lobo	Wolf Spider
5	Genus *Coryphasia*	Papa-Moscas	Jumping Spider
6	Genus *Enoploctenus*	Aranha-Errante	Wandering Spider
7	Genus *Phiale*	Papa-Moscas	Jumping Spider
8	Genus *Phoneutria*	Aranha-Armadeira	Brazilian Wandering Spider
9	Genus *Selenops*	Aranhas-Murais	Wall Spiders
10	Genus *Thaumasia*	Aranha-Pescadora	Fishing Spider
11	Genus *Trechalea*	Aranha-da-Cachoeira	Waterfall Spider
12	Genus *Vinnius*	Papa-Moscas	Jumping Spider
13	*Goniosoma unicolor*	Opilião	Harvester
14	*Lasiodora benedeni*	Aranha-Tarântula	Tarantula Spider
15	*Mesabolivar cyaneotaeniatus*	Aranha-Pernalonga	Long-Legged Spider
16	*Mischonyx squalidus*	Opilião	Harvester
17	*Parasteatoda tepidariorum*	Aranha-Doméstica	House Spider
18	*Tityus costatus*	Escorpião-Amarelo	Yellow Scorpion
19	*Trichonephila clavipes*	Aranha-de-Teia-Dourada	Golden Silk Spider

**Table 5:** List of all insect species identified in this study in the Atlantic Forest of southeast Brazil.

	Species name	Popular name (in Portuguese)	Popular name (in English)
1	*Adelpha cocala*	Borboleta-Irmã-Laranja-Lavada	Orange-washed Sister Butterfly
2	*Adelpha cytherea*	Borboleta-Irmã-de-Bandas-Lisas	Smooth-banded Sister Butterfly
3	*Aedes aegypti*	Mosquito-da-Dengue	Dengue Mosquito
4	*Agelaia angulata*	Vespa-Social	Social Wasp
5	*Ameris ynca*	Broca-da-Ráquis-Foliar	Leaf-Rachis Borer
6	*Anartia amathea*	Borboleta-Pavão-Escarlate	Scarlet Peacock Butterfly
7	*Apis mellifera*	Abelha-Do-Mel	Honey Bee
8	*Apistosia judas*	Mariposa-Judas	Judas Moth
9	*Apogeshna stenialis*	Mariposa	Moth
10	*Apoica gelida*	Vespa	Wasp
11	*Argia indicatrix*	Libélula	Dragonfly
12	*Arsenura armida*	Borboleta	Butterfly
13	*Ascalapha odorata*	Mariposa-Bruxa	Witch Moth
14	*Asphaera hilaris*	Besouro-Saltador	Jumping Beetle
15	*Astylus antis*	Besouro	Beetle
16	*Battus polystictus*	Borboleta-Rabo-de-Andorinha	Swallowtail Butterfly
17	*Bombus brasiliensis*	Abelhão	Bumblebee
18	*Bothynus entellus*	Besouro-Escaravelho	Scarbug Beetle
19	*Caligo brasiliensis*	Borboleta-Coruja	Owl Butterfly
20	*Caligo illioneus*	Borboleta-Coruja	Owl Butterfly
21	*Camponotus rufipes*	Sarassará-de-Pernas-Ruivas	Red-legged Warbler
22	*Camponotus sericeiventris*	Formiga-Dourada	Golden Ant
23	*Cardioscarta albigutta*	Cigarrinha	Cicada
24	*Carineta diardi*	Cigarra Colorida	Colorful Cicada
25	*Castoraeschna januaria*	Libélula	Dragonfly
26	*Catacantha ferruginea*	Mariposa	Moth
27	*Cephalotes atratus*	Formiga	Ant
28	*Cerastipsocus venosus*	Piolho de Tronco	Barklouse
29	*Ceretes thais*	Mariposa	Moth
30	*Cerodirphia rosacordis*	Mariposa	Moth
31	*Chauliognathus flavipes*	Besouro-Soldado	Soldier Beetle
32	*Chauliognathus missionum*	Besouro-Soldado	Soldier Beetle
33	*Chlorida costata*	Besouro-Serra-Pau	Longhorn Beetle
34	*Chloropteryx opalaria*	Mede-Palmos	Inchworm
35	*Chromacris speciosa*	Gafanhoto-Soldado	Soldier Grasshopper
36	*Chydarteres dimidiatus*	Besouro-Serra-Pau	Longhorn Beetle
37	*Cissia eous*	Mariposa	Moth
38	*Cladoxerus cryphaleus*	Bicho-Pau	Stick Insect
39	*Clinodiplosis profusa*	Mosquito-de-Galha	Gallfly
40	*Compsus niveus*	Gorgulho-de-Nariz-Largo	Broad-nosed Weevil
41	*Condica mobilis*	Mariposa	Moth
42	*Copaxa decrescens*	Mariposa	Moth
43	*Ctenoscelis acanthopus*	Besouro-Serra-Pau	Longhorn Beetle
44	*Diabrotica limitata*	Besouro-Vaquinha	Cow Beetle
45	*Diaethria clymena*	Borboleta-Oitenta-e-Oito	Eighty-Eight Butterfly
46	*Diaethria eluina*	Borboleta-Oitenta-e-Oito	Eighty-Eight Butterfly
47	*Discodon cinctum*	Besouro-Soldado	Soldier Beetle
48	*Dismorphia crisia*	Borboleta-Branca-Mimética	Mimetic White Butterfly
49	*Doxocopa laurentia*	Borboleta-Mal-Casados	Turquoise Emperor Butterfly
50	*Dynastor darius*	Borboleta-Coruja	Owl Butterfly
51	*Ectima thecla*	Borboleta	Butterfly
52	*Enantia lina*	Borboleta-Mímica-Branca	White Mime Butterfly
53	*Epiphile orea*	Borboleta	Butterfly
54	*Episcada striposis*	Borboleta-Asa-de-Vidro	Glass-Wing Butterfly
55	*Erythrodiplax melanorubra*	Libélula	Dragonfly
56	*Eteona tisiphone*	Borboleta	Black Earwig Butterfly
57	*Euborellia annulipes*	Tesourinha-Preta	Yellow Ghost Butterfly
58	*Eurema albula*	Borboleta-Fantasma-Amarela	Yellow Mimosa Butterfly
59	*Eurema nise*	Borboleta-Mimosa-Amarela	Brown Butterfly
60	*Forsterinaria quantius*	Borboleta-Marrom	Brown Butterfly
61	*Gonodonta pyrgo*	Borboleta-Perfuradora-de-Fruta	Fruit-Borer Butterfly
62	*Hamadryas arete*	Borboleta-Pororó-Azul	Blue Pororó Butterfly
63	*Hamadryas epinome*	Borboleta-Pororó	Pororó Butterfly
64	*Helias phalaenoides*	Borboleta	Butterfly
65	*Heliconius erato*	Borboleta-da-Flor-da-Paixão-Vermelha	Red Passionflower Butterfly
66	*Heliconius ethilla*	Borboleta-Maria-Boba	Silly Mary Butterfly
67	*Heliconius ethilla narcaea*	Borboleta-Maria-Boba	Silly Mary Butterfly
68	*Heliopetes arsalte*	Borboleta	Butterfly
69	*Heraclides anchisiades*	Rosa-de-Luto	Mourning Rose Butterfly
70	*Heraclides androgeus*	Borboleta-Rabo-de-Andorinha	Swallowtail Butterfly
71	*Homalinotus coriaceus*	Broca-Do-Cacho-Do-Coqueiro	Coconut Bunch Borer
72	*Hoplocrates cephalotes*	Formiga-Veludo	Velvet Ant
73	*Hoplomutilla spinosa*	Formiga-Feiticeira	Witch Ant
74	*Hypanartia lethe*	Borboleta	Butterfly
75	*Hypothyris ninonia daeta*	Borboleta-Preta-e-Amarela	Black and Yellow Butterfly
76	*Ithomia agnosia*	Borboleta-Asa-de-Vidro	Glass Wing Butterfly
77	*Ithomia drymo*	Borboleta-Asa-de-Vidro	Glass Wing Butterfly
78	*Junonia evarete*	Borboleta-Olho-de-Pavão-Diurno	Daytime Peacock Eye Butterfly
79	*Largus rufipennis*	Percevejo-Grande	Large Stink Bug
80	*Leptinopterus femoratus*	Besouro	Beetle
81	*Leptoglossus gonagra*	Percevejo-Escuro	Stink Bug
82	*Leptoglossus zonatus*	Percevejo-Gaúcho	Stink Bug
83	*Leptoscelis elongator*	Percevejo	Stink Bug
84	*Lochmaeocles fasciatus*	Besouro-Serra-Pau	Longhorn Beetle
85	*Lonomia obliqua*	Taturana Oblíqua	Oblique Caterpillar
86	*Mecistogaster ornata*	Libélula- Helicóptero-deponta-limão	Lemon-tipped Dragonfly
87	*Megalopyge basalis*	Mariposa	Moth
88	*Melanolestes lugens*	Percevejo-Pirata	Pirate Stink Bug
89	*Melucha phyllocnemis*	Percevejo-Melucha	Melucha Stink Bug
90	*Meroncidius flavolimbatus*	Esperança	Honeymoth
91	*Mnasinous cinnamomea*	Mariposa	Moth
92	*Monecphora cingulata*	Cigarrinha-das-Pastagens	Pasture Cicada
93	*Morpho helenor*	Borboleta Capitão-Do-Mato	Captain Butterfly
94	*Morpho hercules*	Borboleta	Butterfly
95	*Musca domestica*	Mosca-Doméstica	Housefly
96	*Myscelia orsis*	Borboleta	Butterfly
97	*Naupactus auricinctus*	Besouro-Gorgulho	Weevil Beetle
98	*Omophoita octoguttata*	Besouro-Saltador	Jumping Beetle
99	*Omophoita sexnotata*	Besouro-Saltador	Jumping Beetle
100	*Opoptera syme*	Borboleta	Butterfly
101	*Opsiphanes invirae*	Lagarta-Verde-Das-Palmeiras	Green Palm Caterpillar
102	*Oxyagrion pavidum*	Libélula	Dragonfly
103	*Pachylia syces*	Mariposa-Falcão	Falcon Moth
104	*Pantherodes pardalaria*	Mariposa-Onça	Jaguar Moth
105	*Paracles fusca*	Lagarta	Caterpillar
106	*Paratype univitta*	Mariposa	Moth
107	*Parides proneus*	Borboleta	Butterfly
108	*Pelecinus polyturator*	Vespa	Wasp
109	*Pero ogmopoea*	Mariposa	Moth
110	*Phenax variegata*	Cigarra	Cicada
111	*Phoebis neocypris*	Borboleta-Gema	Butterfly
112	*Phoebis philea*	Borboleta-Gema	Butterfly
112	*Photina vitrea*	Louva-a Deus	Praying Mantis
114	*Pierella nereis*	Borboleta	Butterfly
115	*Polybia ignobilis*	Capuxu	Hooded Butterfly
116	*Praepedaliodes phanias*	Borboleta-Duende	Elf Butterfly
117	*Prisopus villosipes*	Bicho-Pau	Stick Insect
118	*Pyralis manihotalis*	Mariposa	Moth
119	*Quesada gigas*	Cigarra-Gigante	Giant Cicada
120	*Renia sobrialis*	Mariposa	Moth
121	*Rhinostomus barbirostris*	Besouro-Broca-do-Estipe	Stem Borer Beetle
122	*Rhionaeschna planaltica*	Libélula	Dragonfly
123	*Ronderosia bergii*	Gafanhoto	Grasshopper
124	*Scaptotrigona bipunctata*	Tubuna	Tubuna Beetle
125	*Siproeta epaphus trayja*	Borboleta	Butterfly
126	*Sphenorhina rubra*	Cigarrinha-das-Pastagens	Pasture Cicada
127	*Spicauda teleus*	Borboleta	Butterfly
128	*Stictochilus tripunctatus*	Percevejo	Trunk Bug
129	*Synargis paulistina*	Borboleta-Paulistinha	Butterfly
130	*Synoeca cyanea*	Marimbondo-Tatu	Tatu Wasp
131	*Taeniotes amazonum*	Besouro-Tartaruga	Turtle Beetle
132	*Taygetis mermeria*	Mariposa	Moth
133	*Tegosa claudina*	Borboleta	Butterfly
134	*Telegonus alector*	Borboleta-de-duas-Barras	Two-Barred Butterfly
135	*Temenis laothoe*	Borboleta-Vermelha-Tomate	Tomato-Red Butterfly
136	*Tetragonisca angustula*	Jataí-Amarela	Yellow Jataí Beetle
137	*Themesia auricapilla*	Besouro-da-Folha	Leaf Beetle
138	*Thyrinteina arnobia*	Mariposa	Moth
139	*Trigona spinipes*	Abelha-Irapuã	Irapuã Bee
140	*Trypoxylon albitarse*	Vespa	Wasp
141	*Urbanus proteus*	Borboleta-Lagarta-das-Folhas	Leaf Caterpillar Butterfly
142	*Vilerna rugulosa*	Gafanhoto-de-Antenas-Curtas	Short-Antennaed Grasshopper
143	*Xanthopastis timais*	Mariposa-Espanhola	Spanish Moth
144	*Xylophanes tersa*	Esfinge-Tersa	Sphinx Moth
145	*Zammara tympanum*	Cigarra-Verde	Green Cicada
146	*Zelurus circumcinctus*	Percevejo-Predador	Predatory Bug

**Table 6:** List of all plant species identified in this study in the Atlantic Forest of southeast Brazil.

	Species name	Popular name (in Portuguese)	Popular name (in English)	Introduced
1	*Aechmea ramosa*	Bromélia	Bromeliad	
2	*Alpinia zerumbet*	Gengibre-Concha	Variegated Ginger	X
3	*Alternanthera brasiliana*	Penicilina	Ruby Leaf	
4	*Amblystegium serpens*	Musgo	Creeping Moss	
5	*Amphilophium crucigerum*	Pente-de-Macaco	Monkey’s Hairbrush	
6	*Ananas comosus bracteatus*	Abacaxi-Vermelho	Red Pineapple	
7	*Ancistrotropis robusta*	Feijão-Selvagem	Fabaceae	
8	*Antigonon leptopus*	Amor-Agarradinho	Coral Vine	X
9	*Apeiba tibourbou*	Pau-de-Jangada	Tobago Sandbox	
10	*Araucaria angustifolia*	Araucária	Candelabra Tree	
11	*Archontophoenix cunninghamiana*	Palmeira-Real	King Palm	X
12	*Artocarpus heterophyllus*	Jaca	Jackfruit	X
13	*Baccharis trimera*	Carqueja-Verdadeira	Bitter Broom	
14	*Begonia cucullata*	Azedinha-Do-Brejo	Wax Begonia	
15	*Bidens rubifolia*	Picão-Amarelo	Beggarticks	
16	*Billbergia amoena*	Guaricana-Uva	Bromeliad	
17	*Brasiliopuntia brasiliensis*	Urumbeba	Brazilian Prickly Pear	
18	*Browallia americana*	Brovália	Bush Violet	
19	*Brugmansia suaveolens*	Trombeta	Angel’s Trumpet	X
20	*Brunfelsia hydrangeiformis*	Manacá-de-Jardim	Solanaceae	
21	*Brunfelsia pauciflora*	Manacá-Graúdo	Solanaceae	
22	*Brunfelsia uniflora*	Manacá-de-Cheiro	Manaca	
23	*Caamembeca oxyphylla*	Caamembeca	Polygalaceae	
24	*Calliandra eriophylla*	Esponjinha	Fairy Duster	
25	*Calliandra haematocephala*	Esponja-Vermelha	Powder Puff Plant	
26	*Calliandra tweediei*	Topete-de-Cardeal	Flamebush	
27	*Callisia repens*	Dinheiro-Em-Penca	Turtle Vine	
28	*Cariniana estrellensis*	Jequitibá-Branco	Plains Oak	
29	*Cassia fistula*	Cássia-Imperial	Golden Shower Tree	X
30	*Catasetum cernuum*	Orquídea	Orchid	
31	*Catasetum integerrimum*	Orquídea	Orchid	
32	*Cattleya forbesii*	Orquídea	Orchid	
33	*Cecropia glaziovii*	Embaúba-Vermelha-Do-Sul	Red Cecropia	
34	*Cecropia hololeuca*	Embaúba-Prateada	Silver-Leaf Cecropia	
35	*Ceiba speciosa*	Paineira	Silk Floss Tree	
36	*Centropogon cornutus*	Aninga	Campanulaceae	
37	*Chlorophytum comosum*	Clorofito	Spider Plant	X
38	*Chromolaena laevigata*	Falso-Cambará	Asteraceae	
39	*Cissus alata*	Cipó-Uva	Grape Ivy	
40	*Citrus medica*	Cidreira	Citron	
41	*Clethra scabra*	Pau-de-Cinzas	Rough-Leaved Clethra	
42	*Commelina obliqua*	Trapoeraba	Dayflower	
43	*Comparettia coccinea*	Orquídea-Aia	Orchid	
44	*Cordia alliodora*	Lourinho	Spanish Elm	
45	*Cordyline fruticosa*	Dracena Vermelha	Ti Plant	X
46	*Costus spiralis*	Cana-Do-Brejo	Corkscrew Ginger	
47	*Croton urucurana*	Sangra-d’Água	Dragon’s Blood Tree	
48	*Ctenanthe oppenheimiana*	Maranta-Tricolor	Tiger Stripe Plant	
49	*Cupania vernalis*	Camboatá-Vermelho	Sapindaceae	
50	*Cyperus luzulae*	Capim-de-Botão	Rush-Like Flatsedge	
51	*Cyrtocymura scorpioides*	Erva-de-São-Simão	Asteraceae	
52	*Dahlia pinnata*	Dália-de-Jardim	Garden Dahlia	
53	*Desmodium incanum*	Carrapicho-Beiço-de-Boi	Creeping Beggarweed	X
54	*Dichorisandra thyrsiflora*	Gengibre-Azul	Blue Ginger	
55	*Dietes grandiflora*	Moréia	Large Wild Iris	X
56	*Dillenia indica*	Maçã-de-Elefante	Elephant Apple	X
57	*Echinodorus grandiflorus*	Chapéu-de-Couro	Cornstalk Dracaena	
58	*Eranthemum pulchellum*	Camarão Azul	Blue Eranthemum	X
59	*Eriobotrya japonica*	Nespereira	Loquat	X
60	*Erythrina speciosa*	Mulungu-Do-Litoral	Ivory Coral Tree	
61	*Eucalyptus tereticornis*	Eucalipto-Vermelho-da-Mata	Forest Red Gum	
62	*Eugenia uniflora*	Pitangueira	Brazilian cherry	
63	*Euphorbia pulcherrima*	Poinsétia	Poinsettia	X
64	*Euterpe edulis*	Juçara	Assai Palm	
65	*Galium hypocarpium*	Saco-de-Touro	Orange-fruited bedstraw	
66	*Geonoma schottiana*	Guaricana	Arecaceae	
67	*Gibasis pellucida*	Véu-de-Noiva	Tahitian bridalveil	
68	*Gomesa flexuosa*	Chuva-de-Ouro	Orchid	
69	*Gomesa planifolia*	Orquídea	Orchid	
70	*Gomphrena celosioides*	Perpétua-Branca	Gomphrena-Weed	
71	*Handroanthus chrysotrichus*	Ipê-Do-Morro	Golden Trumpet Tree	
72	*Hedychium coccineum*	Gengibre-Vermelho	Orange Ginger Lily	X
73	*Hedychium coronarium*	Lírio-Do-Brejo	Butterfly Ginger Lily	X
74	*Heliconia farinosa*	Caeté	Heliconiaceae	
75	*Heliconia psittacorum*	Helicônia-Papagaio	Heliconiaceae	X
76	*Heliconia spathocircinata*	Helicônia	Heliconiaceae	
77	*Impatiens walleriana*	Beijo-Turco	Busy Lizzie	X
78	*Ipomoea indica*	Bons-Dias	Oceanblue Morning Glory	
79	*Jacaranda mimosifolia*	Jacarandá-Mimoso	Blue Jacaranda	X
80	*Lantana camara*	Camará-Juba	Shrub Verbena	X
81	*Ludwigia elegans*	Cruz-de-Malta	Water Primrose	
82	*Magnolia grandiflora*	Magnólia-Branca	Southern Magnolia	
83	*Manettia luteorubra*	Trepadeira-de-Flor- Amarela-Vermelha	Brazilian Firecracker	
84	*Mangifera indica*	Mangueira	Indian Mango	X
85	*Melastoma malabathricum*	Rododendro	Malabar Melastome	
86	*Miconia albicans*	Canela-de-Velho	Melastomataceae	
87	*Miconia australis*	Pixirica	Melastomataceae	
88	*Miconia calvescens*	João-Henrique	Melastomataceae	
89	*Miconia melastomoides*	Pixirica	Melastomataceae	
90	*Miconia theizans*	Jacatirão	Melastomataceae	
91	*Musa × paradisiaca*	Banana	Plantain	X
92	*Myrcia splendens*	Batinga-Roxa	Punchberry	
93	*Myrsine coriacea*	Capororoca	Leathery Colicwood	
94	*Oxalis corniculata*	Erva-Azeda	Creeping Woodsorrel	X
95	*Oxalis debilis*	Azeda	Largeflower pink-sorrel	
96	*Palicourea longipedunculata*	Erva-de-Rato	Poison Herb	
97	*Palicourea marcgravii*	Cafezinho	Poison Herb	
98	*Passiflora speciosa*	Maracujá- Ornamental	Passifloraceae	
99	*Pavonia sepium*	Amarelinha	Shell Flower	
100	*Peltophorum dubium*	Angico-Canjiquinha	Horsebush	
101	*Persea americana*	Abacate	Avocado	X
102	*Phlebodium aureum*	Polipódio-Dourado	Golden Polypody	
103	*Phlebodium pseudoaureum*	Samambaia-Azul	False Golden Polypody	
104	*Pilea microphylla*	Brilhantina	Artillery-Plant	X
105	*Piper anisum*	Erva-Doce	Piperaceae	
106	*Piptadenia gonoacantha*	Pau-Jacaré	Fabaceae	
107	*Pleroma arboreum*	Quaresmeira-Branca	Brazilian Glorytree	
108	*Pleroma granulosum*	Quaresmeira	Glory Bush Tree	
109	*Pleroma heteromallum*	Orelha-de-Onça	Princess Flower	
110	*Pleroma urvilleanum*	Orelha-de-Onça	Princess Flower	X
111	*Plinia cauliflora*	Jaboticabeira	Brazilian Grapetree	
112	*Plinia edulis*	Cambucá	Myrtaceae	
113	*Portea petropolitana*	Gravatá	Bromeliad	
114	*Pouteria caimito*	Abiu	Abiu	
115	*Pseudogynoxys chenopodioides*	Senécio Confuso	Flame Vine	
116	*Psidium cattleyanum*	Araçá	Guava	
117	*Pyrostegia venusta*	Flor-de-São-João	Flame Vine	
118	*Rhynchospora corymbosa*	Capim-Navalha	Golden Beaksedge	
119	*Roupala montana*	Carvalho-Brasileiro	Roupale	
120	*Rubus rosifolius*	Amora-Vermelha	roseleaf Bramble	X
121	*Salvia splendens*	Flor-de-Cardeal	Scarlet Sage	
122	*Scutellaria uliginosa*	Escutelária	Skullcap	
123	*Selaginella doederleinii*	Musgo-Tapete	Doederlein’s Spikemoss	X
124	*Selenicereus setaceus*	Pitaya-Tiú	Cactaceae	
125	*Selenicereus undatus*	Rainha-da-Noite	Night-Blooming Cereus	X
126	*Senna spectabilis*	Cássia-Do-Nordeste	Whitebark Senna	
127	*Sida planicaulis*	Mata-Pasto	Spiny-head Sida	
128	*Solanum didymum*	Jurubeba	Nightshade	
129	*Spermacoce verticillata*	Vassourinha-de-Botão	Shrubby False Buttonweed	
130	*Sphagneticola trilobata*	Margaridão	Daisy	
131	*Stromanthe thalia*	Caeté-Roxo	Marantaceae	
132	*Syagrus romanzoffiana*	Jerivá	Queen Palm	
133	*Syngonium angustatum*	Singônio	Arrowhead Vine	X
134	*Syzygium jambos*	Jambeiro-Branco	Rose Apple	X
135	*Tara spinosa*	Falso Pau-Brasil	Spiny Holdback	
136	*Tetrapterys mucronata*	Tetrapiteres	Woody Vine	
137	*Tillandsia stricta*	Cravo-Do-Mato	Upright Airplant	
138	*Tradescantia fluminensis*	Erva-da-Fortuna	Small-leaf Spiderwort	
139	*Trimezia liebmannii*	Falsa-Íris-Esplêndida	Walking Iris	
140	*Trimezia sabini*	Falsa-Íris-Roxa	Walking Iris	
141	*Triumfetta semitriloba*	Carrapicho	Sacramento Bur	
142	*Vismia guianensis*	Lacre	Wax Tree	
143	*Vriesea gigantea*	Bromélia-Gigante	Bromeliad	
144	*Vriesea procera*	Bromélia-Do-Alto	Bromeliad	
145	*Youngia japonica*	Barba-de-Falcão	Oriental Hawksbeard	X
146	*Zantedeschia aethiopica*	Jarro	Arum Lily	X

**Table 7:** List of all amphibians, mollusks and crustacea species identified in this study in the Atlantic Forest of southeast Brazil.

Mollusca				
	Species name	Popular name (in Portuguese)		Popular name (in English)
1	*Antidrymaeus interpunctus*	Caramujo-Terrestre		Land snail
2	*Bahiensis punctatissimus*	Caramujo-Terrestre		Land snail
3	Subfamília Scolodontinae	Caramujo-Terrestre		Land snail
**Crustaceans**				
**Species name**		**Popular name** **(in Portuguese)**		**Popular name** **(in English)**
*Trichodactylus fluviatilis*		Caranguejo-de-Água-Doce		Freshwater Crab
**Amphibians**				
	**Species name**	**Popular name** **(in Portuguese)**	**Popular name** **(in English)**	**Endemic**
1	*Boana pardalis*	Rã-Leopardo	Leopard Frog	X
2	*Dendropsophus anceps*	Sapo-Estrela	Star Frog	X
3	*Dendropsophus minutus*	Perereca-Pequena	Small Tree Frog	
4	*Haddadus binotatus*	Rã-da-Mata	Forest Frog	X
5	*Itapotihyla langsdorffii*	Perereca-Castanhola	Brown Tree Frog	
6	*Proceratophrys boiei*	Sapo-de-Chifre- Sulista	Southern Horned Frog	X
7	*Rhinella icterica*	Cururu-Amarelo	Yellow Toad	
8	*Rhinella ornata*	Sapo-de-Floresta	Forest Frog	X
9	*Thoropa miliaris*	Rã-do-Costão	Coastal Frog	X

**Table 8:** List of all reptile species identified in this study in the Atlantic Forest of southeast Brazil.

	Species name	Popular name (in Portuguese)	Popular name (in English)
1	*Aspronema dorsivittatum*	Lagarto-Liso	White-lined Skink
2	*Bothrops jararaca*	Jararaca-da-Mata	Jararaca Snake
3	*Chironius bicarinatus*	Cobra-Cipó-Verde	Green Vine Snake
4	*Chironius exoletus*	*Caninana-Verde*	Green Whipsnake
5	*Dryophylax nattereri*	Cobra-Corre-Campo	Coastal House Snake
6	*Enyalius brasiliensis*	Camaleãozinho	Little Chameleon
7	*Enyalius perditus*	Camaleão-Papa-Vento	Wind-Eater Chameleon
8	*Erythrolamprus aesculapii*	Bacorá	South American False Coralsnake
9	*Hemidactylus mabouia*	Lagartixa-Doméstica- Tropical	Tropical House Lizard
10	*Hydromedusa maximiliani*	Cágado-de-Serra	Mountain Turtle
11	*Ophiodes striatus*	Cobra-de-Vidro-Verde	Green Glass Snake
12	*Oxyrhopus clathratus*	Cobra-Falsa-Coral	False Coral Snake
13	*Salvator merianae*	Teiú-Gigante	Giant Tegu
14	*Tropidurus torquatus*	Tropiduro-Comum	Amazon Lava Lizard
15	*Xenodon neuwiedii*	Boipeva-da-Mata	Forest Boipeva

**Table 9:** List of all mammal species identified in this study in the Atlantic Forest of southeast Brazil.

	Species name	Popular name (in Portuguese)	Popular name (in English)
1	*Cabassous tatouay*	Tatu-de-Rabo- Mole-Grande	Greater naked-tailed armadillo
2	*Callithrix aurita*	Sagui-da-Serra-Escuro	Buffy-tufted marmoset
3	*Callithrix aurita × jacchus*	Sagui-híbrido	Hybrid Marmoset
4	*Callithrix aurita × penicillata*	Sagui-híbrido	Hybrid Marmoset
5	*Callithrix penicillata*	Sagui-de-Tufos-Pretos	Black-tufted Marmoset
6	*Cavia aperea*	Preá-Brasileiro	Brazilian Guinea Pig
7	*Cerdocyon thous*	Cachorro-Do-Mato	Crab-eating fox
8	*Coendou spinosus*	Coandu-Alaranjado	Paraguaian hairy dwarf porcupine
9	*Cuniculus paca*	Paca	Lowland paca
10	*Dasypus novemcinctus*	Tatu-Galinha	Nine-banded Armadillo
11	*Dasypus septemcinctus ssp. septemcinctus*	Tatu-Mirim	Seven-banded armadillo
12	*Didelphis aurita*	Gambá-de-Orelha- Preta	Big-eared opossum
13	*Eira barbara*	Irara	Tayra
14	*Galictis cuja*	Furão-Pequeno	Lesser grison
15	*Leopardus guttulus*	Gato-do-Mato-Pequeno	Southern tigrina
16	*Nasua nasua*	Quati-de-Cauda- Anelada	South American coati
17	*Procyon cancrivorus*	Guaxinim-Sul- Americano	Crab-eating raccoon
18	*Puma concolor*	Puma	Puma
19	*Sciurus brasiliensis ssp. ingrami*	Caxinguelê	Ingram’s squirrel
20	*Sylvilagus brasiliensis*	Tapiti	Common tapeti

**Fig 3:**
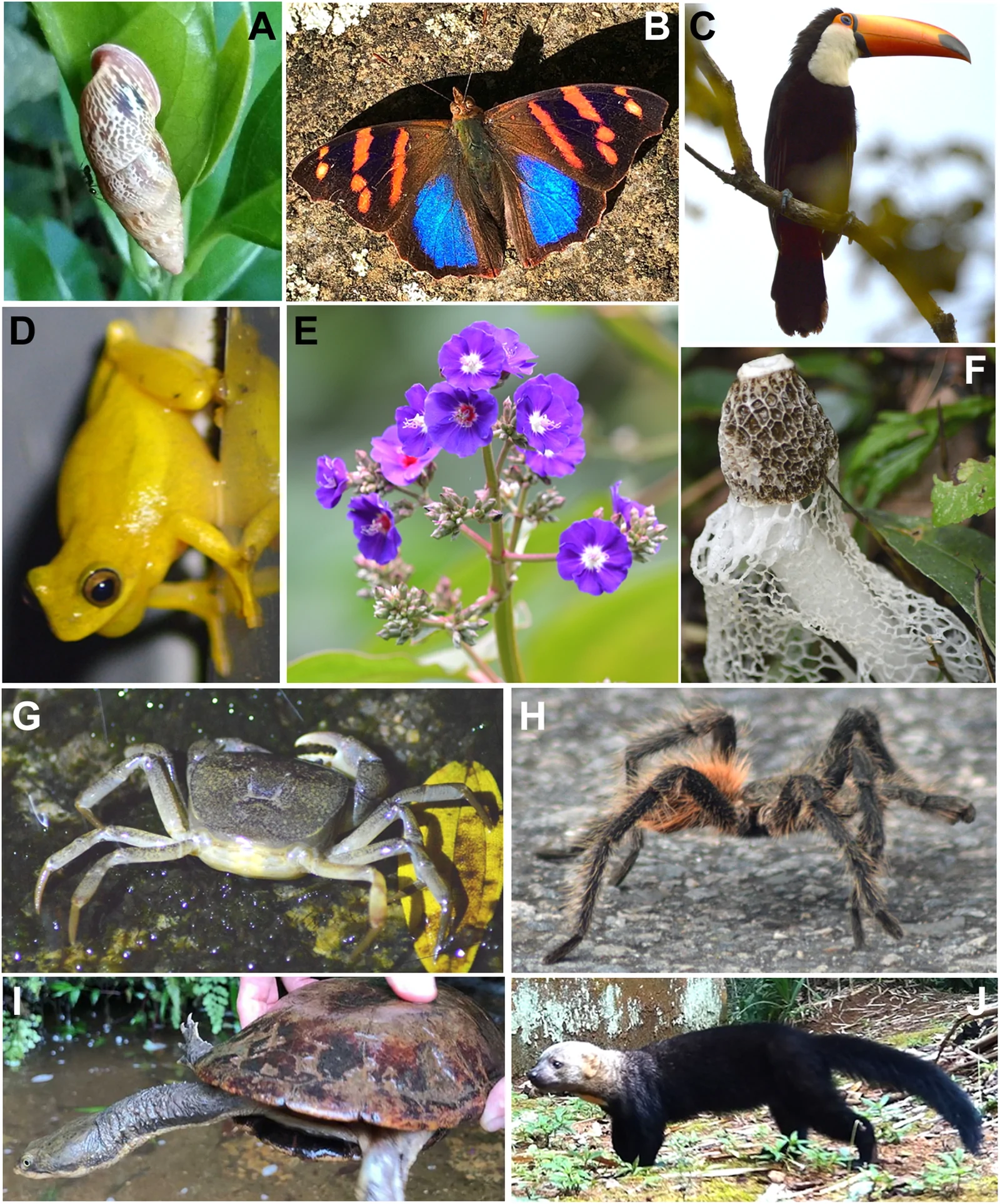
Biodiversity in the Atlantic Forest of southeast Brazil. One example of each of the ten (10) groups of living organisms is represented: Mollusca (*Bahiensis punctatissimus* in **A**), Insects (*Epiphile orea* in **B**), Birds (*Ramphastos toco* in **C**), Amphibians (*Dendropsophus minutus* in **D**), Plants (*Pleroma heteromallum* in **E**), Fungi (*Phallus indusiatus* in **F**), Crustacea (*Trichodactylus fluviatilis* in **G**), Arachnida (*Lasiodora benedeni* in **H**), Reptilia (*Hydromedusa maximiliani* in **I**), Mammals (*Eira barbara* in **J**).

**Fig 4:**
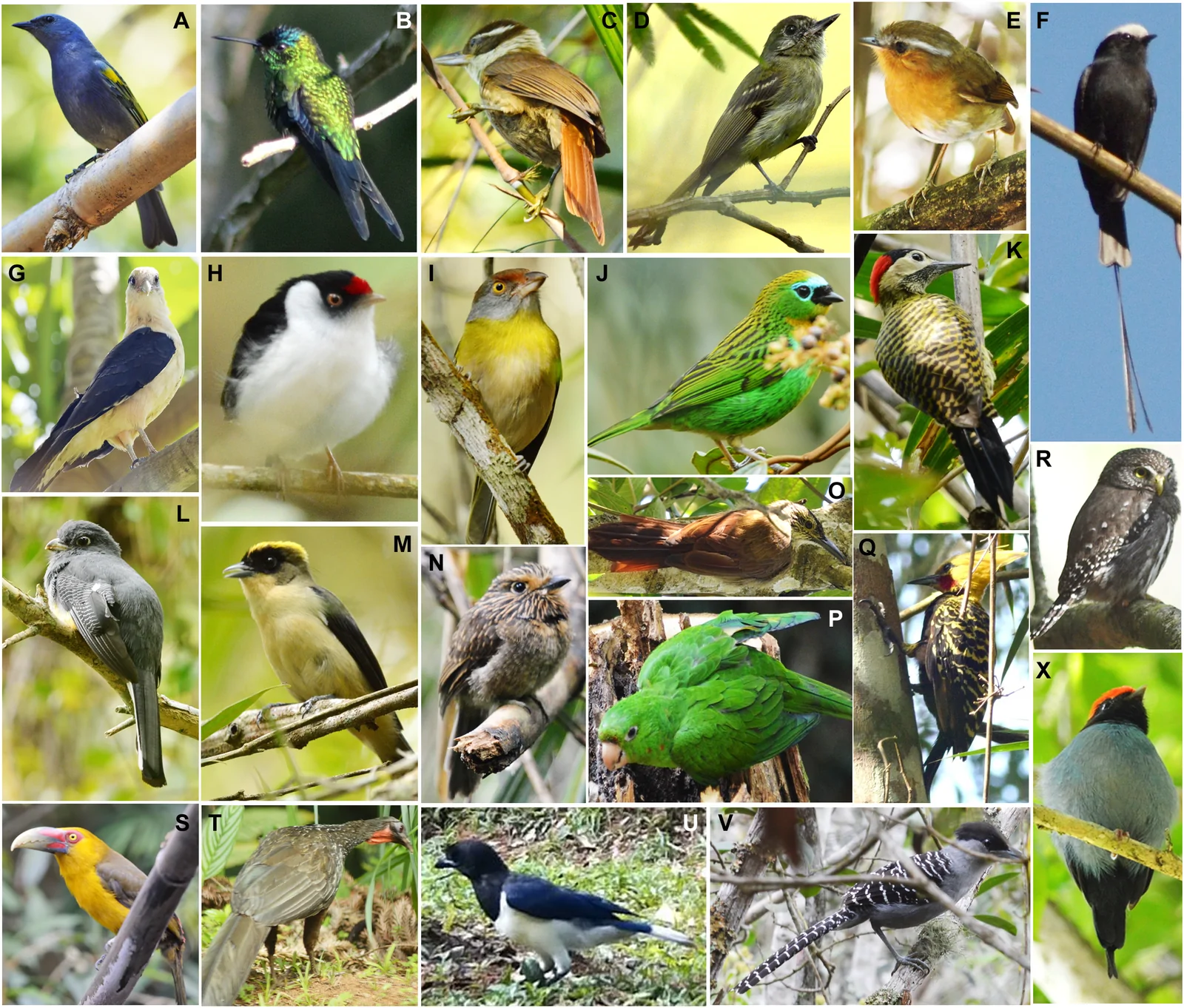
Biodiversity of birds in the Atlantic Forest of southeast Brazil. Twenty three (23) examples of birds are shown: *Thraupis ornata* (**A**), *Thalurania glaucopis* (**B**), *Anabazenops fuscus* (**C**), *Leptopogon amaurocephalus* (**D**), *Conopophaga lineata* (**E**), *Colonia colonus* (**F**), *Daptrius chimachima* (**G**), *Ilicura militaris* (**H**), *Cyclarhis gujanensis* (**I**), *Tangara desmaresti* (**J**), *Colaptes melanochloros* (**K**), *Trogon surrucura* (**L**), *Trichothraupis melanops* (**M**), *Malacoptila striata* (**N**), *Dendrocolaptes platyrostris* (**O**), *Psittacara leucophthalmus* (**P**), *Celeus flavescens* (**Q**), *Glaucidium brasilianum* (**R**), *Pteroglossus bailloni* (**S**), *Penelope obscura* (**T**), *Cyanocorax cristatellus* (**U**), *Batara cinerea* (**V**), *Chiroxiphia caudata* (**X**).

**Fig 5:**
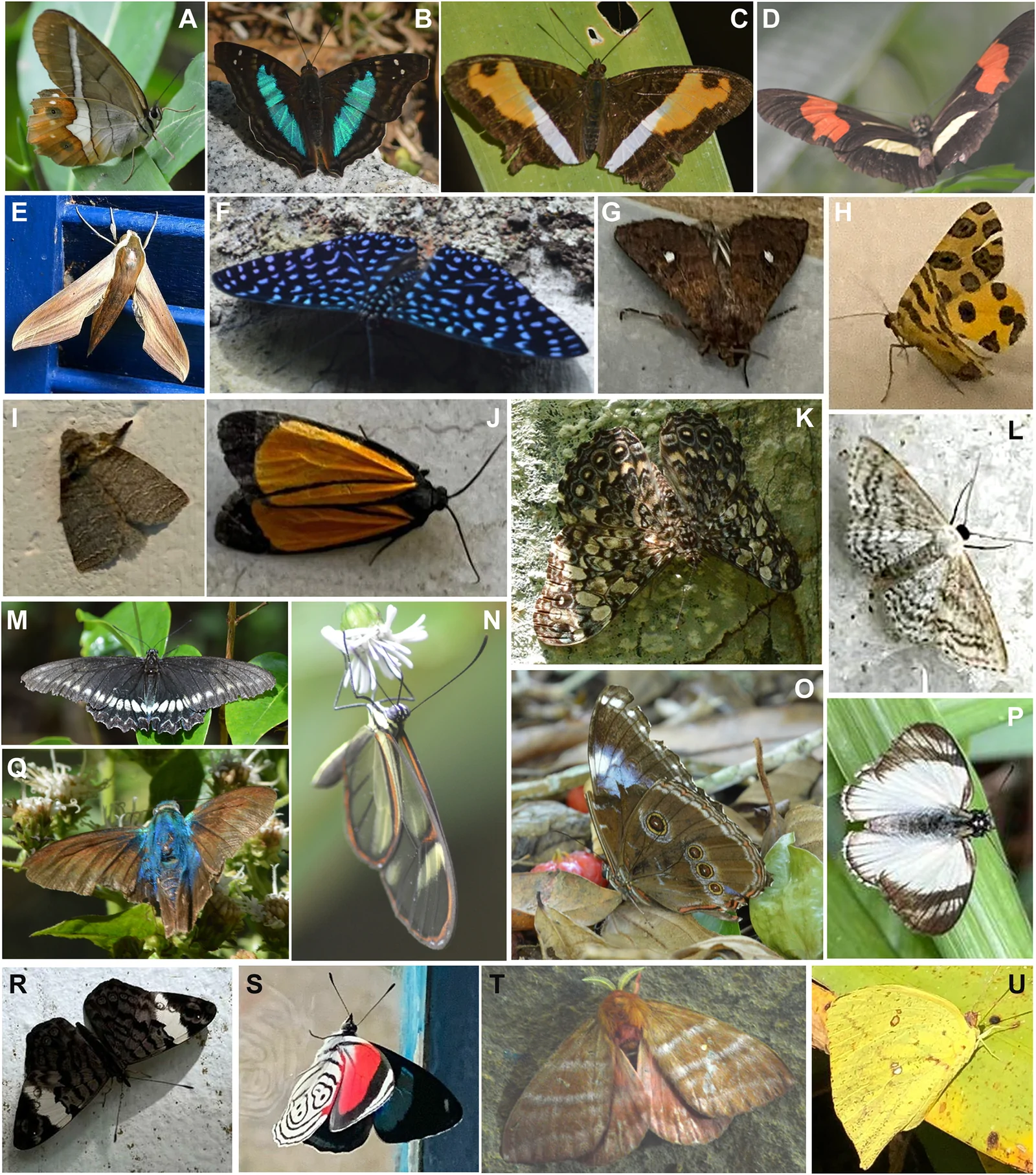
Biodiversity of Lepidoptera in the Atlantic Forest of southeast Brazil. Twenty one (21) examples of lepidoptera are shown: *Pierella nereis* (**A**), *Doxocopa laurentia* (**B**), *Adelpha cocala* (**C**), *Heliconius erato ssp. Phyllis* (**D**), *Xylophanes tersa* (**E**), *Hamadryas arete* (**F**), *Condica mobilis* (**G**), *Pantherodes pardalaria* (**H**), Genus *Meragisa* (**I**), Genus *Scea* (**J**), *Hamadryas epinome* (**K**), *Chloropteryx opalaria* (**L**), *Battus polystictus* (**M**), *Episcada striposis* (**N**), *Morpho helenor* (**O**), *Heliopetes arsalte* (**P**), *Telegonus alector* (**Q**), *Ectima thecla* (**R**), *Diaethria eluina* (**S**), *Cerodirphia rosacordis* (**T**), *Phoebis neocypris* (**U**).

Some of our iNaturalist data were incorporated into other databases, such as in Global Biodiversity Information Facility (GBIF, 950 occurrences) and Sistema de Informação sobre a Biodiversidade Brasileira (SiBBr, https://sibbr.gov.br/). These occurrences can be found at “GBIF.org (1 September 2026) GBIF Occurrence Download https://doi.org/10.15468/dl.mm8bxs”, available at https://www.gbif.org/occurrence/search?recordedBy=claudia_mermelstein&geometry=POLYGON%28%28-43.01459+-22.2552%2C-43.01838+-22.2581%2C-43.01303+-22.26484%2C-43.00942+-22.26167%2C-43.01459+-22.2552%29%29). These Citizen Science platforms increase the availability of our data.

One of the strengths of our work was the use of high-quality images of living organisms for identification and classification. Figs 3–5 show examples of these images. It has been claimed that photographic images are essential to modern zoology, functioning as non-invasive tools for research, documentation, and conservation. In contrast, in a recent work we reported that the field of zoology does not frequently use photographic images in research papers for the description of animal species characterization [18]. Contrary to records based only on an individual person observation, images are a permanent record of the observation event, shareable with the community for posterior analysis. The same applies to the recording of sound, which is a permanent and shareable observation.

Insects and plants were by far the groups with the largest number of species identified, followed by birds, fungi and arachnids (Fig 4 and Tables 2–4). Interestingly, birds were the group with the largest number of individual observations (Fig 4 and Table 2), followed by insects and plants (Fig 5 and Tables 5–6). Crustaceans and mollusks were the groups with the smallest number of observations and species, with only two mollusca species of land snails identified (*Antidrymaeus interpunctus* and *Bahiensis punctatissimus*) and only one crustacea specie (*Trichodactylus fluviatilis*), a freshwater crab (Fig 3 and Table 7). Different groups of animals differ in several characteristics, including their size, color, habitat and behavior, and therefore different approaches are necessary to acquire their images and classify the observations in species.

### Birds

The region studied has an impressive variety of birds. Most of them were easily observed, and usually we were able to produce quality photos, since they were registered with telephoto lenses during the day (Fig 4). They show a large behavior repertoire, appearing in different habitat conditions (denser or sparser vegetation, for instance). Interestingly, there seems to be more identifiers of birds in the iNaturalist application, compared to all other animal groups. This observation can be explained by the high popularity of birds among citizens in general, and particularly among amateur citizens [19]. Bird watching is a democratic hobby that can be done anywhere, from a backyard to remote wilderness, and requires little initial investment.

A total of 99 species of birds have been recorded and identified in our study in the Brejal (Petrópolis, RJ, Brazil) area (Table 2). Tonetti and colleagues [20] compiled 326 bird species in another Atlantic Forest region in Serra da Cantareira (São Paulo, SP, Brazil), in a much larger area and during a longer time period compared to our study.

Birds were the only group in which we used three different software, iNaturalist, Merlin and Addax, for classification (Fig 4 and Table 2). The majority of the bird species were identified through images, but some birds (15 species) were identified only through their sounds, using Merlin, since they were not easily observed. These birds are *Crypturellus obsoletus* (brown tinamou), *Drymophila ochropyga* (ochre-rumped antbird), *Lochmias nematura* (sharp-tailed streamcreeper), *Lurocalis semitorquatus* (short-tailed nighthawk), *Myiozetetes similis* (social flycatcher), *Procnias nudicollis* (bare-throated bellbird), *Saltator maximus* (buff-throated saltator), *Saltator similis* (green-winged saltator), *Sclerurus scansor* (rufous-breasted leaftosser), *Syndactyla rufosuperciliata* (buff-browed foliage-gleaner), S*pizaetus tyrannus* (black hawk-eagle), *Strix virgata* (mottled owl), *Thraupis sayaca* (sayaca tanager), *Tyranus melancholicus* (tropical kingbird), and *Zonotrichia capensis* (rufous-collared sparrow). Several possibilities can explain this difficulty, such as that they are well camouflaged within the vegetation, that their frequency in number of individuals is lower compared to other birds, and that they are less tolerant of human contact.

Another fascinating behavior that we witnessed and registered was the complex sounds produced by the two bird species *Cyanocorax cristatellus* (curl-crested jay) and *Psarocolius decumanus* (crested oropendola). These birds engage complex and varied songs and sounds for social bonding, territorial defense, and attracting mates [21]. Males often incorporate sounds from other birds, imitating them, probably to display their ability to learn, increasing their chances of breeding success.

Furthermore, we observed different species of Passeriformes close together, forming mixed-species flocks [22], in specific areas, suggesting a possible protective group behavior for benefits like better predator detection (more eyes/ears). One example of these mixed-bird species flocks we recorded with Merlin was composed of: *Basileuterus culicivorus* (golden-crowned warbler), *Chiroxiphia caudata* (blue manakin), *Synallaxis ruficapilla* (rufous-capped spinetail), *Cyclarhis gujanensis* (rufous-browed peppershrike), and *Thamnophilus caerulescens* (variable antshrike).

It was easy to notice differences in the geographic and spatial distribution of birds within the area of study. Some birds were more frequently observed in areas close to water, or in areas with more direct sunshine, or hidden within bushes, or in very high trees, or in tree hollows, or on the ground. Therefore, it was essential in our study to observe birds in all these different habitats, in different hours of the day, and all year round (including summer, autumn, winter and spring seasons).

Since we found 100 species of birds in an area of approximately 50,000 square meters, we can estimate 1 bird species for each 500 square meters.

### Insects

We observed 147 insect species, which comprise the largest number of observed species in our study. Since insects are amongst a larger number of species in nature, with orders of magnitude more than vertebrates, the relative number of insect species we obtained does not correspond to nature. This is probably due both to the difficulty in observation (due to their small size), collection (they usually require nets) and identification of insect species. In fact, we were surprised that lepidoptera (butterflies and moths) correspond to 50% of all the insect species observed and identified (Fig 6 and Table 5). We can hypothesize that: (i) lepidoptera are in fact highly frequent in these high-altitude tropical forests, (ii) most of the butterflies are easily spotted during the day (because of their beautiful impacting colors) and moths are easily spotted during the nights (because of their light-attraction behavior in areas near human habitations), and (iii) lepidoptera are well known taxonomically [23,24] and they are easily classified in iNat. These explanations are not mutually exclusive, and we believe that they all had an impact on our data. In the Charles Darwin book, The Voyage of the Beagle (1845), he says “The large and brilliantly colored lepidoptera bespeak the zone they inhabit, far more plainly than any other race of animals” in relation to the exuberant colors of the butterflies of the Atlantic forest of Rio de Janeiro [25].

**Fig 6:**
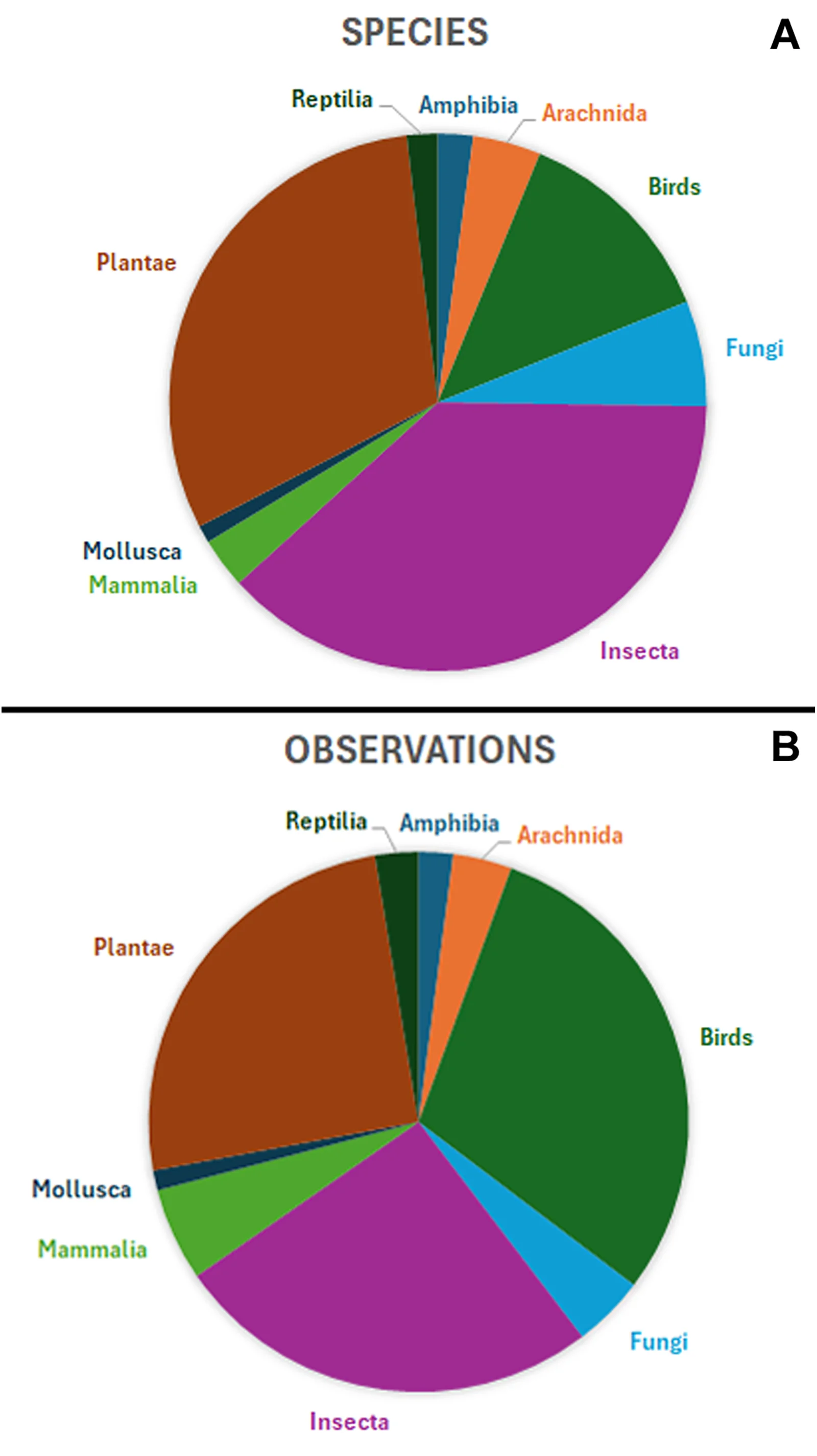
Frequency of species and observations registered in iNaturalist. Pie charts showing the frequencies of species (**A**) and observations (**B**) registered in iNaturalist application of the groups of living organisms described in this study. Only one species of crustacea (*Trichodactylus fluviatilis*) was found in this area of study and therefore crustacea was not represented in the charts.

### Mammals

Most of the mammals were registered with camera traps. In fact, most of them were never seen in any other way. We observed 19 mammal species (Table 9), not including bats and rodents, which are difficult to photograph. Seventeen species were observed in a nearby protected area using camera traps [26], 12 of which were also found in our study; 5 were not observed in our area and 3 were recorded in our region but not in their study. Besides bats and rodents, several other mammals are known to exist in the Atlantic forest but were not observed. Some, like the jaguar (*Panthera onca*), are known to be extinct in the area, while others are known to exist in the vicinity but have not been observed by either of the studies ([26] and our present study), such as agouti (*Dasyprocta*), capybara (*Hydrochoerus hydrochaeris*) and sloth (*Bradypus variegatus*).

The observation that caused a great surprise in this study was the presence of the large predator mountain lion (*Puma concolor*) in the Brejal region (Table 9). Puma (“suçuarana”, mountain lion or “onça parda”) individuals were detected only with night vision trail cameras. Several observations of Puma were registered and identified in iNat. Pumas are under the “near threatened (NT)” conservation status in Brazil (IUCN Red List), which highlights the importance of our study towards the conservation of this area and this feline species (Table 11). Although Brejal still has a large area of forest, it also contains several small farms with human presence, and therefore, the presence of Puma can be harmful and of high concern for both humans and felines. Pumas, who are wide-ranging mammals, could be travelling from the nearby Parque Municipal Montanhas de Teresópolis, where they have already been described [26]. This kind of observation highlights the importance of this and other nearby protected areas as reservoirs of biodiversity. This also suggests that privately owned properties can contribute to mosaics together with formally protected areas.

Another important piece of information that we gather from our study is that besides *Callithrix aurita*, an endangered marmoset, we also identified in the studied area the presence of hybrids of marmoset *Callithrix aurita* × *Callithrix jacchus*, and *Callithrix aurita* × *Callithrix penicillata* (Table 9). *C. jacchus* and *C. penicillata* are invasive species in the Atlantic Forest of southeastern Brazil, heavily impacting native biodiversity. Introduced via illegal trade, they thrive in disturbed habitats and edge forests. They pose a significant threat to the native, endangered marmoset *C. aurita* through competition, habitat displacement, and, most critically, hybridization, leading to genetic erosion [27]. The classification of marmoset hybrids is an example of the importance of community-based CS. Several iNat identifiers helped to establish the species and hybrids status, which was beyond the capability of AI-automated classification.

One weakness of our study was the absence of observations of bats (Chiroptera). Since we used only non-invasive methods (photographs, videos and sound recording) to observe animals, we were not able to photograph bats. Bats are very hard to photograph because they are usually nocturnal and fly very fast. Collecting bats and other mammals in nature traditionally employs specialized equipment like fine-mesh mist nets, which can unfortunately inflict distress on animals [28]. Although we did not collect any Chiroptera, we were able to see bats several times flying in the area of study at sunset. Cronemberger and colleagues [29] described the presence of twenty-three species of Chiroptera in the Serra dos Órgãos National Park (Teresópolis and Petrópolis, RJ, Brazil), a nearby preserved and large Atlantic forest biome. Another group of mammals that is under-represented in our study are rodents, which are considered difficult to observe and classify due to their high diversity and small morphological differences [30]. Although we recorded rodents with camera traps, we could not classify them on the basis solely of the image, and we did not include them.

### Plants

We observed several types of plants, ranging in size from small flowering plants to large trees, almost 2 meters in diameter. Plants can be difficult to classify due to insufficient occurrence data [31]. Feeley (2015) estimated that 74% of the plant species in South America had fewer than 20 records and 10% lacked any record at all, known as invisible species [32]. Furthermore, to precisely classify plants it is important to register flowers, leaves and seeds, which can be difficult to be accomplished for tall trees. Several of the plants we observed have been introduced in the region by anthropomorphic ways (Table 6). Most of these introduced plants are fructiferous, ornament and flowering plants. These findings were anticipated since Brejal is an area with extensive human presence, even though it has several preserved forest areas nearby, contiguous with less preserved areas (Fig 1). Brazil has numerous introduced plants, some of which can be invasive, impacting native ecosystems by outcompeting local flora. Among the invasive plants that are present in large numbers in Brejal are the lilies *Alpinia zerumbet* (shell ginger), *Hedychium coccineum* (orange ginger lily), *Hedychium coronarium* (white ginger lily), which are all well-adapted to marsh areas and compete for space with local marsh plants.

### Camera traps

Interestingly, the trail cameras allowed us to identify twenty-four (24) species, ten of which were only observed with these camera traps (Table 10). These ten species are *Cerdocyon thous* (crab-eating fox), *Crypturellus obsoletus* (brown tinamou), *Cuniculus paca* (spotted lowland paca), *Dasypus novemcinctus* (nine-banded armadillo), *Eira Barbara* (tayra), *Leopardus guttulus* (southern tiger cat), *Nasua nasua* (south American coati), *Procyon cancrivorus* (crab-eating raccoon), *Puma concolor* (mountain lion), *Sylvilagus brasiliensis* (Brazilian cottontail rabbit). Among the 23 species observed with camera traps, the most frequently seen were *Aramides saracura* (slaty-breasted wood rail), *Cuniculus paca* (spotted lowland paca), *Didelphis aurita* (black-eared opossum) and *Penelope obscura* (dusky-legged guan), in this order of frequency (Fig 7). Several studies have shown that camera traps are invaluable tools for the observation of elusive species without the disturbance of human presence [33]. In our study, these devices were set up in locations without human presence and left unattended for several days. Thanks to their discreet design and low light imaging capability they were able to capture images of animals that otherwise would not be seen. We did not use small animal traps (boxes) for the collection of animals in our study, since they are invasive forms of animal analysis.

**Table 10:** List of all species observed with night trap cameras in the Atlantic Forest of southeast Brazil.

	Species name	Popular name (in Portuguese)	Popular name (in English)	IUCN status	Animal group
1	*Aramides saracura*	Saracura-Do-Mato	Slaty-Breasted Wood-Rail		Bird
2	*Baryphthengus ruficapillus*	Juruva	Rufous-Capped Motmot		Bird
3	*Cerdocyon thous*	Cachorro-do-Mato	Crab-Eating-Fox		Mammal
4	*Cavia aperea*	Preá-Brasileiro	Brazilian Guinea Pig		Mammal
5	*Callithrix aurita × penicillata*	Sagui	Marmoset		Mammal
6	*Crypturellus obsoletus*	Inhambuguaçu	Brown-Tinamou		Bird
7	*Cuniculus paca*	Paca	Spotted-Paca		Mammal
8	*Cyanocorax cristatellus*	Gralha-do-Campo	Curl-Crested-Jay		Bird
9	*Dasypus novemcinctus*	Tatu-Galinha	Nine-Banded- Armadillo		Mammal
10	*Didelphis aurita*	Gambá-de-Orelha- Preta	Black-Eared- Opossum		Mammal
11	*Eira barbara*	Irara	Tayra		Mammal
12	*Leopardus guttulus*	Gato-Do-Mato- Pequeno	Southern-Tiger Cat	VU	Mammal
13	*Leptotila verreauxi*	Juriti-Pupu	White-Tipped Dove		Bird
14	*Morpho helenor*	Borboleta Capitão-Do-Mato	Common-Blue- Morpho		Insect
15	*Morpho hercules*	Borboleta-Hércules	Hercules-Morpho		Insect
16	*Nasua nasua*	Quati-de-Cauda- Anelada	Ring-Tailed-Coati		Mammal
17	*Penelope obscura*	Jacuguaçu	Dusky-Legged Guan		Bird
18	*Procyon cancrivorus*	Guaxinim-Sul- Americano	South American Raccoon		Mammal
19	*Puma concolor*	Puma	Puma	NT	Mammal
20	*Rupornis magnirostris*	Gavião-Carijó	Roadside-Hawk		Bird
21	*Salvator merianae*	Teiú-Gigante	Giant-Tegu		Reptile
22	*Sciurus brasiliensis*	Caxinguelê	Brazilian-Squirrel		Mammal
23	*Sylvilagus brasiliensis*	Tapiti	Brazilian- Cottontail	EN	Mammal
24	*Turdus rufiventris*	Sabiá-Laranjeira	Rufous-bellied Thrush		Bird

**Fig 7:**
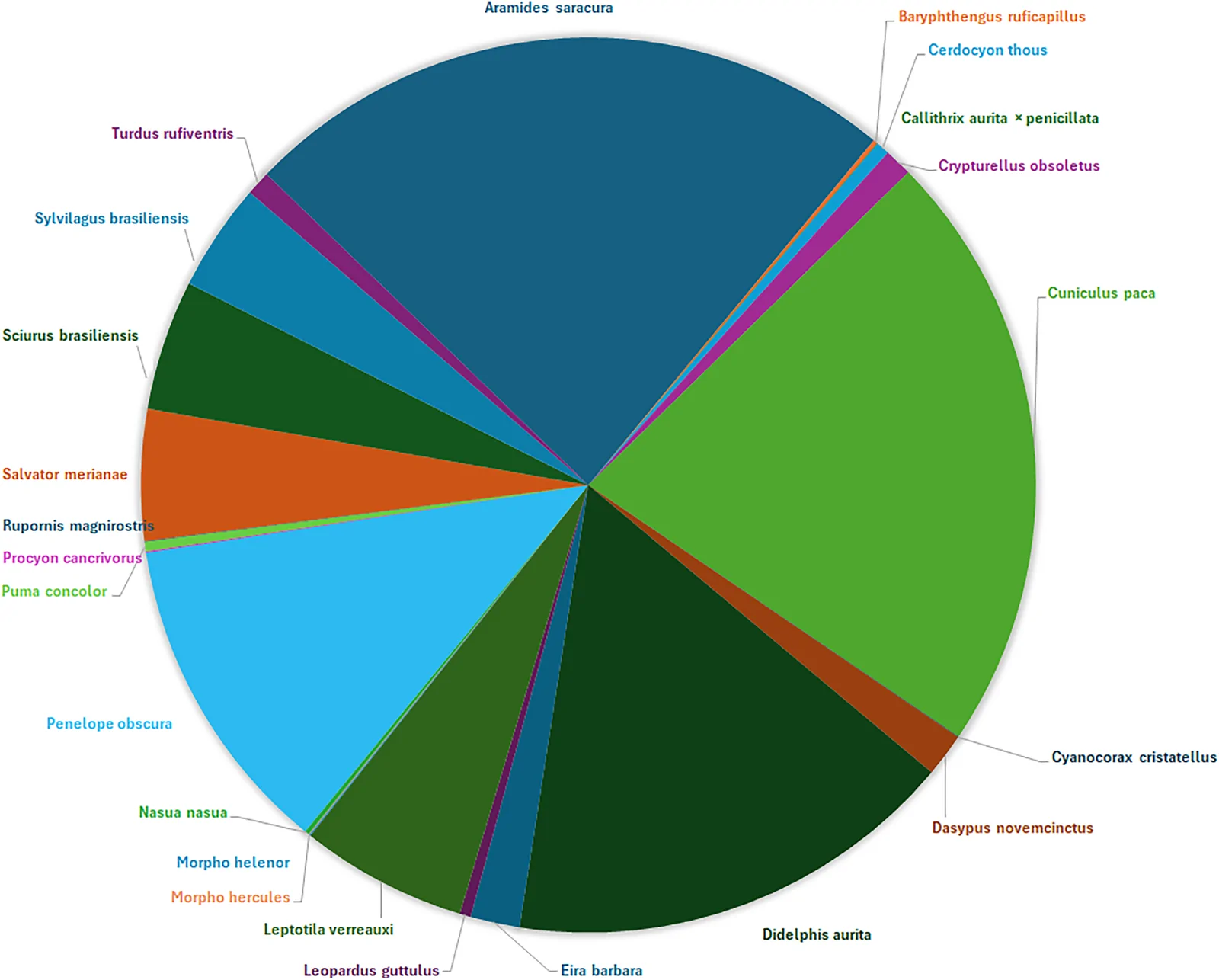
Frequency of animal species captured by camera traps. Among the twenty-three species observed with night vision cameras, the most frequently seen on the night cameras were *Aramides saracura* (slaty-breasted wood rail), *Cuniculus paca* (spotted lowland paca), *Didelphis aurita* (black-eared opossum) and *Penelope obscura* (dusky-legged guan), in this order of frequency.

One interesting behavior that the camera traps witnessed was opossums (*Didelphis aurita*) carrying leaves in their prehensile tails. This interesting behavior allows opossums to insulate and prepare their nests in small underground caves. By grabbing materials with their mouth, passing them to their front feet, then to their back feet, they curl their tail over the bundle to carry it like a fifth limb [34].

Camera traps generated more than 10,000 pictures of animals with 54 GigaBytes of memory in our 6-year survey. They also generated 5,730 videos, which we did not include in this manuscript since they cannot be submitted to iNaturalist nor are easily analyzed by animal identification software. We used AddaxAI software with AI-driven computer vision models to help us to classify animals and to find and eliminate images without animals (blank images), which were nearly 4,000 images. After that, we manually corrected the misidentifications of the Addax software and ended up with a total of 3,800 picture files. Addax was important to save us time and efficiency in the analysis of images.

Domestic dogs and cats were registered by the camera traps in our study, but we decided to not include them in the list of mammals found in this region.

### Threatened species

In our study, we found 12 species (4 plants, 4 mammals, 3 birds, and 1 reptile, Table 11) considered threatened by IUCN (The International Union for Conservation of Nature, https://www.iucnredlist.org/). These species are *Araucaria angustifolia* (Brazilian pine tree), *Brugmansia suaveolens* (Brazilian white angel’s trumpet plant), *Callithrix aurita* (buffy-tufted marmoset), *Hydromedusa maximiliani* (Brazilian snake-necked turtle), *Jacaranda mimosifolia* (tree), *Leopardus guttulus* (southern tiger cat), *Piculus aurulentus* (white-browed woodpecker), *Plinia edulis* (Cambucá plant), *Primolius maracanã* (macaw), *Pteroglossus bailloni* (saffron toucanet), *Puma concolor* (mountain lion), *Sylvilagus brasiliensis* (Brazilian cottontail rabbit). These results highlight the importance of conservation efforts in the Atlantic Forest.

**Table 11:** List of all threatened species identified in this study in the Atlantic Forest of southeast Brazil.

	Species name	Popular name (in Portuguese)	Popular name (in English)	IUCN status	Animal group
1	*Araucaria angustifolia*	Araucária	Brazilian Pine	CR	Plantae
2	*Brugmansia suaveolens*	Trombeta	Angel’s Trumpet	EW	Plantae
3	*Callithrix aurita*	Sagui-da-Serra- Escuro	Buffy-Tufted- Ear Marmoset	EN	Mammal
4	*Hydromedusa maximiliani*	Cágado-de-Serra	Brazilian Snake-NeckeTurtle	VU	Reptile
5	*Jacaranda mimosifolia*	Jacarandá- Mimoso	Jacaranda Tree	VU	Plantae
6	*Leopardus guttulus*	Gato-Do-Mato- Pequeno	Southern Tiger Cat	VU	Mammal
7	*Piculus aurulentus*	Pica-Pau- Dourado	White-Browed Woodpecker	NT	Bird
8	*Plinia edulis*	Cambucá	Cambucá	VU	Plantae
9	*Primolius maracana*	Maracanã	Macaw-Finch (Spanish Macaw)	NT	Bird
10	*Pteroglossus bailloni*	Araçari-Banana	Saffron Toucanet	NT	Bird
11	*Puma concolor*	Puma	Puma	NT	Mammal
12	*Sylvilagus brasiliensis*	Tapiti	Brazilian Cottontail	EN	Mammal

### Endemic species

It has been described that the Atlantic forest has a high level of endemism. In our study, more than 60% of the amphibian species were endemic (Table 7). The endemic amphibian species are *Boana pardalis* (leopard tree frog), *Dendropsophus anceps* (estrella treefrog), *Haddadus binotatus* (clay robber frog), *Proceratophrys boiei* (smooth-horned frog), *Rhinella ornata* (ornate forest toad), and *Thoropa miliaris* (rock river frog). Brejal is very humid and has a vast area of waterlogged soil and small rivers, which are ideal for the amphibian’s ecosystem. These unique endemic amphibian species are known to be extremely sensitive to environmental changes, highlighting the importance of conservation efforts, as many are threatened by factors like pollution and climate change.

### Animals living near humans’ dwellings

Finally, we found several animal species living in the houses near the forest, showing an adaptation to living in or around human dwellings. These animals are often drawn to residential areas due to the availability of food and space for their nests. The animals that we identified in nearby houses were *Psittacara leucophthalmus* (white-eyed parakeet), *Pygochelidon cyanoleuca* (blue-and-white swallow), *Musca domestica* (housefly), ants of the Genus *Camponotus* (carpenter ant), *Parasteatoda tepidariorum* (common house spider), *Hemidactylus mabouia* (tropical house gecko), and *Tropidurus torquatus* (neotropical ground lizard). Interestingly, *Psittacara leucophthalmus* and *Pygochelidon cyanoleuca* were frequently found nesting and roosting in crevices in house roofs.

### Classification difficulties

The proportion of observations reaching the species level of classification (considered as Research Grade in the iNaturalist platform) was amphibians (67%), arachnids (45%), birds (98%), fungi (20%), insects (40%), mammals (95%), mollusks (70%), plants (40%), reptiles (87%) and crustaceans (100%), pointing to differences in species identification of these groups. It is clear that some living groups are easier to classify than others. Arachnids, fungi, insects and plants are among groups with less species identified. However, we decided to include some of the observations of these four groups classified at the Genus level (Table 4), because we believe that even this partial information can be useful. In iNaturalist, unidentified species refer to organisms that were not identified to the species level due to a lack of expert knowledge, poor photo quality, or because the species were not yet described by science. It is important to consider the huge differences in species numbers in each group. For instance, there are hundreds of thousands of species of insects already described and many more new species expected to be identified. It is obvious that these differences impact the number of known species and, consequently, the proportion of identifiable species [35]. Studies like ours could encourage other scientists and citizens to explore these neglected species in the Atlantic forest of southeast Brazil.

### Potential caveats of our study

A common definition of citizen science (CS) is the public participation in scientific research, where non-scientists collect data and share observations, aiming to advance science education. Another definition accounts for citizens (including scientists not trained in a given field) to produce scientific relevant data [36]. In our study, only the two authors gathered data, analyzed the information and shared them with the global community of naturalists. Although we are scientists, we do not work with zoology, botany or ecology, but with cell biology. Importantly, the data that we up-loaded in the iNaturalist and other CS platforms were analyzed and classified by experts in specific living organisms. These experts include scientists and non-scientists in an effort to improve species classification.

We assume that our study qualifies as CS because: 1) We show that it is feasible and easy for citizen scientists to assess the biodiversity in small public and private properties; 2) The study was conceived and conducted by scientists that have been trained in a different field; 3) Citizen Science communities such as Merlin and iNaturalist participated in the classification; 4) The list of species in an area is a powerful educational and scientific tool.

One of the main goals of CS is to increase science awareness. Even though we are educated in general biology concepts, through our work in this study we became much more involved in learning about our local species diversity. There was a strong emotional involvement in finding out and registering the amazing diversity literally in our backyard.

A common criticism about CS is the low quality of data generated by non-professional scientists [37]. We consider that this is not our case, since there were several objective and rigorous limits to include each of our records, including analysis by peers. Furthermore, we consider that some types of information can be quite simple and yet highly relevant, such as the recorded observation of a given species in a particular area. Since species classification can be interpretative and can change with time, it is arguable that the records are actually the permanent and relevant data. It is worth mentioning that lists of species in a given area are important for a variety of applications [38].

We conducted our study in a privately owned property. Most biodiversity assessments are conducted in public conservation areas. We hope that the feasibility of our approach would incentivize similar assessments in other private properties.

## Conclusions

In conclusion, here we analyzed the biodiversity of an Atlantic forest region in southeast Brazil, and we found 492 species of animals, fungi and plants, many of which are endemic, threatened and/or understudied. We were able to collect and describe a huge amount of data in terms of images, sounds and animal behavior. We have taken advantage of easy-to-use mobile phones, camera traps, photographic cameras equipped with tele-macro lenses, and AI software, such as iNaturalist, Merlin and Addax (Table 1). Our work shows how feasible it is to study the biodiversity of a specific region using simple hardware and software devices. As emphasized by Zheng and colleagues [39], the high sampling coverage and rapid data accumulation achieved by Citizen Science platforms have the potential to revolutionize biodiversity monitoring. Importantly, we obtained data related to all living organisms found in the analyzed region, and not only to a specific group of organisms (for example, insects). Thus, one of the greater strengths of our study was the qualitative analysis of all the observed living species, which will permit further quantitative studies focused on the ecological interconnections among these organisms. Therefore, we hope that this work can encourage other naturalists, amateur scientists and citizens to expand our knowledge on biodiversity of specific biomes around the world.

## Supporting information

S1 File Table with all iNat records. The table contains all records from the “Brejal-Petrópolis-RJ and its Biodiversity” Project downloaded on December 27, 2025, corresponding to a 6 year-survey (2020–2025). The table contains all amphibians, arachnids, birds, crustaceans, insects, mammals, mollusks and reptiles’ observations registered in the area, including for each observation, the location, time, the species classification and the link to the iNaturalist register.
(XLSX)
