# Supplementary material for: Integrating images, sounds, citizen science and AI to assess the biodiversity of the Atlantic Forest of Brazil
Source: PLoS One. 2026 Oct 7;21(10):e0360070. doi: 10.1371/journal.pone.0360070 (PMC13645092; doi:10.1371/journal.pone.0360070)
Supplement: Supplementary file 1 [file pone.0360070.s003.docx]

Rio de Janeiro, August 23, 2026.

Dear Dr. **Daniel de Paiva Silva**,

We submitted the manuscript "**Integrating images, sounds, citizen science and AI to assess the biodiversity of the Atlantic Forest of Brazil**” (**PONE-D-26-15035**) by Costa et al., for publication in **Plos One**. The reviewers recommended major revisions to the manuscript. We thank the Reviewers for their careful and appropriate analysis. We included a point-by-point response to the reviewer’s comments, which were useful to improve the data presentation and interpretation. The detailed corrections are as follows.

**Journal Requirements:**

1. When submitting your revision, we need you to address these additional requirements.

Please ensure that your manuscript meets PLOS ONE's style requirements, including those for file naming. The PLOS ONE style templates can be found at

<https://journals.plos.org/plosone/s/file?id=wjVg/PLOSOne_formatting_sample_main_body.pdf> and

<https://journals.plos.org/plosone/s/file?id=ba62/PLOSOne_formatting_sample_title_authors_affiliations.pdf>

**Author’s response:** We thank the editor for this comment. We apologize for submitting with the wrong format, and we adjusted the style accordingly.

2. In your Methods section, please provide additional information regarding the permits you obtained for the work. Please ensure you have included the full name of the authority that approved the field site access and, if no permits were required, a brief statement explaining why.

**Author’s response:** We thank the reviewer for this important remark. In fact, we own the property, and therefore no authorization was needed. We changed the text to make this information clear.

3. Please note that PLOS One has specific guidelines on code sharing for submissions in which author-generated code underpins the findings in the manuscript. In these cases, we expect all author-generated code to be made available without restrictions upon publication of the work. Please review our guidelines at <https://journals.plos.org/plosone/s/materials-and-software-sharing#loc-sharing-code> and ensure that your code is shared in a way that follows best practice and facilitates reproducibility and reuse.

**Author’s response:** We did not generate any code; we only used the mentioned software.

4. We note that the grant information you provided in the ‘Funding Information’ and ‘Financial Disclosure’ sections do not match.

When you resubmit, please ensure that you provide the correct grant numbers for the awards you received for your study in the ‘Funding Information’ section.

**Author’s response:** We corrected this.

5. Thank you for stating in your Funding Statement:

This work was supported by grants from Conselho Nacional de Desenvolvimento Científico e Tecnológico (CNPq, funding number 302961/2021-6 to C.M., 308192/2021-4 to M.L.C.) and Fundação de Apoio à Pesquisa do Estado do Rio de Janeiro (FAPERJ, funding number E-26/203.930/2024 to C.M., E26/204.077/2024 to M.L.C.).

Please provide an amended statement that declares *all* the funding or sources of support (whether external or internal to your organization) received during this study, as detailed online in our guide for authors at <http://journals.plos.org/plosone/s/submit-now>. Please also include the statement “There was no additional external funding received for this study.” in your updated Funding Statement.

Please include your amended Funding Statement within your cover letter. We will change the online submission form on your behalf.

**Author’s response:** We thank the editor for this remark, and we corrected the text accordingly.

6. Thank you for stating the following financial disclosure:

This work was supported by grants from Conselho Nacional de Desenvolvimento Científico e Tecnológico (CNPq, funding number 302961/2021-6 to C.M., 308192/2021-4 to M.L.C.) and Fundação de Apoio à Pesquisa do Estado do Rio de Janeiro (FAPERJ, funding number E-26/203.930/2024 to C.M., E26/204.077/2024 to M.L.C.).

Please state what role the funders took in the study. If the funders had no role, please state: "The funders had no role in study design, data collection and analysis, decision to publish, or preparation of the manuscript."

If this statement is not correct you must amend it as needed.

Please include this amended Role of Funder statement in your cover letter; we will change the online submission form on your behalf.

**Author’s response:** We thank the editor for this remark, and we corrected the text accordingly.

7. Thank you for uploading your study's underlying data set. Unfortunately, the repository you have noted in your Data Availability statement does not qualify as an acceptable data repository according to PLOS's standards.

At this time, please upload the minimal data set necessary to replicate your study's findings to a stable, public repository (such as figshare or Dryad) and provide us with the relevant URLs, DOIs, or accession numbers that may be used to access these data. For a list of recommended repositories and additional information on PLOS standards for data deposition, please see <https://journals.plos.org/plosone/s/recommended-repositories>.

**Author’s response:** We thank the editor for this remark. We corrected the manuscript to explain that all data is available in the supplementary file.

8. We note that Figure 1 in your submission contains map and satellite images which may be copyrighted. All PLOS content is published under the Creative Commons Attribution License (CC BY 4.0), which means that the manuscript, images, and Supporting Information files will be freely available online, and any third party is permitted to access, download, copy, distribute, and use these materials in any way, even commercially, with proper attribution. For these reasons, we cannot publish previously copyrighted maps or satellite images created using proprietary data, such as Google software (Google Maps, Street View, and Earth). For more information, see our copyright guidelines: <http://journals.plos.org/plosone/s/licenses-and-copyright>.

We require you to either present written permission from the copyright holder to publish these figures specifically under the CC BY 4.0 license, or remove the figures from your submission:

a. You may seek permission from the original copyright holder of Figure 1 to publish the content specifically under the CC BY 4.0 license.

We recommend that you contact the original copyright holder with the Content Permission Form (<http://journals.plos.org/plosone/s/file?id=7c09/content-permission-form.pdf>) and the following text:

“I request permission for the open-access journal PLOS ONE to publish XXX under the Creative Commons Attribution License (CCAL) CC BY 4.0 (<http://creativecommons.org/licenses/by/4.0/>). Please be aware that this license allows unrestricted use and distribution, even commercially, by third parties. Please reply and provide explicit written permission to publish XXX under a CC BY license and complete the attached form.”

Please upload the completed Content Permission Form or other proof of granted permissions as an "Other" file with your submission.

In the figure caption of the copyrighted figure, please include the following text: “Reprinted from [ref] under a CC BY license, with permission from [name of publisher], original copyright [original copyright year].”

b. If you are unable to obtain permission from the original copyright holder to publish these figures under the CC BY 4.0 license or if the copyright holder’s requirements are incompatible with the CC BY 4.0 license, please either i) remove the figure or ii) supply a replacement figure that complies with the CC BY 4.0 license. Please check copyright information on all replacement figures and update the figure caption with source information. If applicable, please specify in the figure caption text when a figure is similar but not identical to the original image and is therefore for illustrative purposes only.

The following resources for replacing copyrighted map figures may be helpful:

USGS National Map Viewer (public domain): <http://viewer.nationalmap.gov/viewer/>

The Gateway to Astronaut Photography of Earth (public domain): <http://eol.jsc.nasa.gov/sseop/clickmap/>

Maps at the CIA (public domain): <https://www.cia.gov/library/publications/the-world-factbook/index.html> and <https://www.cia.gov/library/publications/cia-maps-publications/index.html>

NASA Earth Observatory (public domain): <http://earthobservatory.nasa.gov/>

Landsat: <http://landsat.visibleearth.nasa.gov/>

USGS EROS (Earth Resources Observatory and Science (EROS) Center) (public domain): [http://eros.usgs.gov/#](http://eros.usgs.gov/)

Natural Earth (public domain): <http://www.naturalearthdata.com/>

**Author’s response:** We thank the editor for this remark, and we prepared a new Figure 1 and a new figure legend. The image of Brazil in Figure 1A was downloaded from a public domain site. Figure 1B was obtained from Instituto Brasileiro de Geografia e Estatística, IBGE, which has an open data policy. Figure 1C was downloaded from Sentinel 2 L2a at Copernicus Sentinel Data and Service Information, which has an open data policy. We added information about the sources in the figure legend.

9. We note that Figures 2, 3 and 4 in your submission contain copyrighted images. All PLOS content is published under the Creative Commons Attribution License (CC BY 4.0), which means that the manuscript, images, and Supporting Information files will be freely available online, and any third party is permitted to access, download, copy, distribute, and use these materials in any way, even commercially, with proper attribution. For more information, see our copyright guidelines: <http://journals.plos.org/plosone/s/licenses-and-copyright>.

We require you to either present written permission from the copyright holder to publish these figures specifically under the CC BY 4.0 license, or remove the figures from your submission:

a. You may seek permission from the original copyright holder of Figures 2, 3 and 4 to publish the content specifically under the CC BY 4.0 license.

We recommend that you contact the original copyright holder with the Content Permission Form (<http://journals.plos.org/plosone/s/file?id=7c09/content-permission-form.pdf>) and the following text:

“I request permission for the open-access journal PLOS ONE to publish XXX under the Creative Commons Attribution License (CCAL) CC BY 4.0 (<http://creativecommons.org/licenses/by/4.0/>). Please be aware that this license allows unrestricted use and distribution, even commercially, by third parties. Please reply and provide explicit written permission to publish XXX under a CC BY license and complete the attached form.”

Please upload the completed Content Permission Form or other proof of granted permissions as an "Other" file with your submission.

In the figure caption of the copyrighted figure, please include the following text: “Reprinted from [ref] under a CC BY license, with permission from [name of publisher], original copyright [original copyright year].”

b. If you are unable to obtain permission from the original copyright holder to publish these figures under the CC BY 4.0 license or if the copyright holder’s requirements are incompatible with the CC BY 4.0 license, please either i) remove the figure or ii) supply a replacement figure that complies with the CC BY 4.0 license. Please check copyright information on all replacement figures and update the figure caption with source information. If applicable, please specify in the figure caption text when a figure is similar but not identical to the original image and is therefore for illustrative purposes only.

**Author’s response:** We thank the editor for this remark. We corrected the copyright requirements of all the images in the iNaturalist platform that we used in the manuscript to comply with the CC BY 4.0 license.

10. Please include captions for your Supporting Information files at the end of your manuscript, and update any in-text citations to match accordingly. Please see our Supporting Information guidelines for more information: <http://journals.plos.org/plosone/s/supporting-information.>

**Author’s response:** We thank the editor for this remark, and we apologize for this mistake. We provided a proper caption for the Supplementary File.

11. If the reviewer’s comments include a recommendation to cite specific previously published works, please review and evaluate these publications to determine whether they are relevant and should be cited. There is no requirement to cite these works unless the editor has indicated otherwise.

**Author’s response:** The reviewers did not recommend to cite specific previously published works**.**

**Additional Editor Comments:**

Dear Dr. Mermelstein,

After the reviews made by independent reviewers, below I indicate the necessary changes you and your co-authors need to do to deliver a new version of your manuscript.

The reviewers recognize this manuscript as a valuable and potentially publishable contribution. The six-year inventory documents substantial biological diversity in a highly threatened Atlantic Forest landscape and demonstrates the usefulness of photography, acoustic recordings, camera traps, digital platforms, and AI-assisted tools for biodiversity monitoring. The resulting dataset has clear potential for conservation, environmental education, and future research. However, substantial revisions are required before the manuscript can be considered for publication.

12. The principal issue concerns the study’s characterization as “citizen science.” The manuscript must clearly explain who collected the observations, how other participants contributed, and how the iNaturalist community supported taxonomic identification and validation.

**Author’s response:** We thank the editor for this comment, which we agree should be clarified. All the observations and data organization were conducted by the authors solely, and no other participants contributed. We included this information in the manuscript. The whole iNat community contributed to the study, through their participation in the identification of species. We incorporated the discussion about CS in the manuscript. We assume the study qualify as CS because: 1) We propose that anyone with access to a private property can conduct a similar study; 2) The study was conceived and conducted by scientists that have been trained in a different area; and 3) Communities of citizens such as Merlin and iNat participated in the classification.

13. The authors should quantify this contribution where possible, including the number of identifiers involved, the proportion of observations reaching Research Grade, and examples of records improved through community review.

**Author’s response:** We thank the editor for these comments. We now include the number of identifiers involved (495) and the proportion of observations reaching Research Grade in the first paragraph of the Results section. We also added an example of a record improved through the iNat community in the “mammals” section of the Results.

14. The manuscript should also explain that eligible Research Grade records are incorporated into databases such as GBIF and SIBBr.

**Author’s response:** We thank the editor for this comment. We incorporated this discussion in the manuscript. In fact, 2000 of our observations were incorporated into the GBIF database. Furthermore, we register on the SIBBr database.

15. A clearer distinction is also needed between AI-generated suggestions and identifications validated by researchers, specialists, or the iNaturalist community. The workflow involving iNaturalist, Merlin, Addax AI, and human verification should be described transparently, together with its limitations. Claims about AI-based citizen science should then be revised to accurately reflect the methods used.

**Author’s response:** We thank the editor for this comment. We agree that the workflow should be clarified. We included this description in the Material and Methods section, and we introduced a new figure to depict it.

16. The taxonomic scope also requires reconsideration. Analyses of native, exotic, endemic, threatened, or invasive status should preferably be restricted to organisms identified at the species level, because genus-level identifications may not permit reliable classification. The authors should clearly report on the taxonomic resolution and validation status of the records included in each analysis.

**Author’s response:** We thank the editor for this comment. We agree that the analyses of status should be restricted to organisms identified at the species level. That is why in our manuscript we only refer to the introduced or threatened status of organisms identified at the species level, including in Table 6 (related to plant species) and Table 11 (related to threatened species).

17. Finally, the ecological and geographical context should be strengthened by describing nearby protected areas and explaining how the study site may contribute to a regional biodiversity corridor. Evidence such as the puma and other wide-ranging mammals may support this interpretation, but conclusions about environmental health, connectivity, or conservation value should remain appropriately cautious.

**Author’s response:** We thank the editor for this comment. We were already mentioning the proximity to protected areas in the description of the place in the methods section. Now we have added a discussion of how the puma could be moving between these areas.

Given the importance of these conceptual and methodological clarifications—particularly because citizen science and AI are central to the title, abstract, and discussion, I recommend Major Revision. A revised manuscript that transparently defines the data-collection and identification workflow, adjusts unsupported claims, and strengthens the regional conservation context, could represent a useful contribution to biodiversity documentation in the Atlantic Forest.

We hope that the modifications made in the new version of the manuscript have properly addressed the criticism and suggestions made by the reviewer, and that the improvements made in the manuscript will be sufficient for its publication in **Plos One**.

We would like to state that all listed authors qualify for authorship and agree with the submission of the revised version of the manuscript. The final version of the manuscript has been seen and approved by all coauthors. The authors declare that they have no conflict of interest.

With kind regards,

Claudia Mermelstein
