# Supplementary material for: Integrating images, sounds, citizen science and AI to assess the biodiversity of the Atlantic Forest of Brazil
Source: PLoS One. 2026 Oct 7;21(10):e0360070. doi: 10.1371/journal.pone.0360070 (PMC13645092; doi:10.1371/journal.pone.0360070)
Supplement: Supplementary file 2 [file pone.0360070.s004.docx]

Rio de Janeiro, September 01, 2026.

Dear Dr. **Daniel de Paiva Silva**,

We submitted the manuscript "**Integrating images, sounds, citizen science and AI to assess the biodiversity of the Atlantic Forest of Brazil**” (**PONE-D-26-15035R1**) by Costa et al., for publication in **Plos One**. The reviewers recommended minor revisions to the manuscript. We thank the Reviewers for their careful and appropriate analysis. We included a point-by-point response to the reviewer’s comments, which were useful for improving the data presentation and interpretation. The detailed corrections are as follows.

**Journal Requirements:**

1 - If the reviewer’s comments include a recommendation to cite specific previously published works, please review and evaluate these publications to determine whether they are relevant and should be cited. There is no requirement to cite these works unless the editor has indicated otherwise.

**Author’s response**: We did not find any recommendations from the reviewers to cite specific previously published works.

2 - Please review your reference list to ensure that it is complete and correct. If you have cited papers that have been retracted, please include the rationale for doing so in the manuscript text, or remove these references and replace them with relevant current references. Any changes to the reference list should be mentioned in the rebuttal letter that accompanies your revised manuscript. If you need to cite a retracted article, indicate the article’s retracted status in the References list and also include a citation and full reference for the retraction notice.

**Author’s response**: We reviewed our reference list, and it is complete and correct. We are not citing retracted papers. We confirmed that by checking all DOI numbers from the cited papers in the software Retraction Watch Database (https://retractiondatabase.org/RetractionSearch.aspx).

**Reviewer #1:**

1 - First, I recommend slightly moderating the use of the phrase “AI-based citizen science approach,” particularly in the Abstract and other prominent sections. Since the field observations were generated solely by the authors, while citizen participation occurred mainly during identification and validation through platforms such as iNaturalist, the current wording may still imply a broader citizen-science data-collection effort than was actually conducted. A more precise formulation might be “an integrated approach combining digital observations, AI-assisted species identification, and citizen-science community validation.” This wording would, in my view, better reflect the actual strengths of the study.

**Author’s response**: We thank the reviewer for this comment. We agree that our paper shows a less conventional approach to Citizen Science. We changed the text in the Abstract according to the reviewer’s suggestions.

2 - Second, please clarify the apparent difference between the 1,515 iNaturalist records downloaded for analysis and the statement that approximately 2,000 observations were incorporated into GBIF. If these numbers refer to different time points, subsets, or database synchronization processes, a brief explanation would be sufficient.

**Author’s response**: We thank the reviewer for this remark. In fact, we were referring to the total number of observations that we registered in iNaturalist that were included in GBif. The actual number of observations in the studied area with iNaturalist that were included in GBif is 950. We corrected the query parameters used in GBif in the text, using the GBif recommended citation format. We included the following new information in the Results section of the manuscript:

Some of our iNaturalist data were incorporated into other databases, such as in Global Biodiversity Information Facility (950 occurrences in “GBIF, GBIF.org (1 September 2026) GBIF Occurrence Download <https://doi.org/10.15468/dl.mm8bxs>”, available at <https://www.gbif.org/occurrence/search?recordedBy=claudia_mermelstein&geometry=POLYGON%28%28-43.01459+-22.2552%2C-43.01838+-22.2581%2C-43.01303+-22.26484%2C-43.00942+-22.26167%2C-43.01459+-22.2552%29%29> ) and Sistema de Informação sobre a Biodiversidade Brasileira (SiBBr, https://sibbr.gov.br/). These Citizen Science platforms increase the availability of our data.

We hope that the modifications made in the new version of the manuscript have properly addressed the criticism and suggestions made by the reviewer, and that the improvements made in the manuscript will be sufficient for its publication in **Plos One**.

We would like to state that all listed authors qualify for authorship and agree with the submission of the revised version of the manuscript. The final version of the manuscript has been seen and approved by all coauthors. The authors declare that they have no conflict of interest.

With kind regards,

Claudia Mermelstein
