# Supplementary material for: Integrating images, sounds, citizen science and AI to assess the biodiversity of the Atlantic Forest of Brazil
Source: PLoS One. 2026 Oct 7;21(10):e0360070. doi: 10.1371/journal.pone.0360070 (PMC13645092; doi:10.1371/journal.pone.0360070)
Supplement: Peer review history

# Peer review: Decision Letter 0

**DOI:** 10.1371/journal.pone.0360070.r001

**Authors:** Daniel Silva

16 Aug 2026

Dear Dr. Mermelstein,

Please submit your revised manuscript by Sep 30 2026 11:59PM. If you will need more time than this to complete your revisions, please reply to this message or contact the journal office at plosone@plos.org. When you're ready to submit your revision, log on to https://www.editorialmanager.com/pone/ and select the 'Submissions Needing Revision' folder to locate your manuscript file.

- A letter that responds to each point raised by the academic editor and reviewer(s). You should upload this letter as a separate file labeled 'Response to Reviewers'.
- A marked-up copy of your manuscript that highlights changes made to the original version. You should upload this as a separate file labeled 'Revised Manuscript with Track Changes'.
- An unmarked version of your revised paper without tracked changes. You should upload this as a separate file labeled 'Manuscript'.

If you would like to make changes to your financial disclosure, please include your updated statement in your cover letter. Guidelines for resubmitting your figure files are available below the reviewer comments at the end of this letter.

If applicable, we recommend that you deposit your laboratory protocols in protocols.io to enhance the reproducibility of your results. Protocols.io assigns your protocol its own identifier (DOI) so that it can be cited independently in the future. For instructions see: https://journals.plos.org/plosone/s/submission-guidelines#loc-laboratory-protocols. Additionally, PLOS ONE offers an option for publishing peer-reviewed Lab Protocol articles, which describe protocols hosted on protocols.io. Read more information on sharing protocols at https://plos.org/protocols?utm_medium=editorial-email&utm_source=authorletters&utm_campaign=protocols.

As the corresponding author, your ORCID iD is verified in the submission system and will appear in the published article. PLOS supports the use of ORCID, and we encourage all coauthors to register for an ORCID iD and use it as well. Please encourage your coauthors to verify their ORCID iD within the submission system before final acceptance, as unverified ORCID iDs will not appear in the published article. *Only* the individual author can complete the verification step; PLOS staff *cannot* verify ORCID iDs on behalf of authors.

We look forward to receiving your revised manuscript.

Kind regards,

Daniel de Paiva Silva, Ph.D.

Academic Editor

PLOS One

**Journal Requirements:**

1. When submitting your revision, we need you to address these additional requirements.

Please ensure that your manuscript meets PLOS ONE's style requirements, including those for file naming. The PLOS ONE style templates can be found at

https://journals.plos.org/plosone/s/file?id=wjVg/PLOSOne_formatting_sample_main_body.pdf and

https://journals.plos.org/plosone/s/file?id=ba62/PLOSOne_formatting_sample_title_authors_affiliations.pdf

2. In your Methods section, please provide additional information regarding the permits you obtained for the work. Please ensure you have included the full name of the authority that approved the field site access and, if no permits were required, a brief statement explaining why.

3. Please note that PLOS One has specific guidelines on code sharing for submissions in which author-generated code underpins the findings in the manuscript. In these cases, we expect all author-generated code to be made available without restrictions upon publication of the work. Please review our guidelines at https://journals.plos.org/plosone/s/materials-and-software-sharing#loc-sharing-code and ensure that your code is shared in a way that follows best practice and facilitates reproducibility and reuse.

4. We note that the grant information you provided in the ‘Funding Information’ and ‘Financial Disclosure’ sections do not match.

When you resubmit, please ensure that you provide the correct grant numbers for the awards you received for your study in the ‘Funding Information’ section.

5. Thank you for stating in your Funding Statement:

This work was supported by grants from Conselho Nacional de Desenvolvimento Científico e Tecnológico (CNPq, funding number 302961/2021-6 to C.M., 308192/2021-4 to M.L.C.) and Fundação de Apoio à Pesquisa do Estado do Rio de Janeiro (FAPERJ, funding number E-26/203.930/2024 to C.M., E26/204.077/2024 to M.L.C.).

Please provide an amended statement that declares *all* the funding or sources of support (whether external or internal to your organization) received during this study, as detailed online in our guide for authors at http://journals.plos.org/plosone/s/submit-now.  Please also include the statement “There was no additional external funding received for this study.” in your updated Funding Statement.

Please include your amended Funding Statement within your cover letter. We will change the online submission form on your behalf.

6. Thank you for stating the following financial disclosure:

This work was supported by grants from Conselho Nacional de Desenvolvimento Científico e Tecnológico (CNPq, funding number 302961/2021-6 to C.M., 308192/2021-4 to M.L.C.) and Fundação de Apoio à Pesquisa

do Estado do Rio de Janeiro (FAPERJ, funding number E-26/203.930/2024 to C.M., E26/204.077/2024 to M.L.C.).

Please state what role the funders took in the study.  If the funders had no role, please state: "The funders had no role in study design, data collection and analysis, decision to publish, or preparation of the manuscript."

If this statement is not correct you must amend it as needed.

Please include this amended Role of Funder statement in your cover letter; we will change the online submission form on your behalf.

7. Thank you for uploading your study's underlying data set. Unfortunately, the repository you have noted in your Data Availability statement does not qualify as an acceptable data repository according to PLOS's standards.

At this time, please upload the minimal data set necessary to replicate your study's findings to a stable, public repository (such as figshare or Dryad) and provide us with the relevant URLs, DOIs, or accession numbers that may be used to access these data. For a list of recommended repositories and additional information on PLOS standards for data deposition, please see https://journals.plos.org/plosone/s/recommended-repositories.

8. We note that Figure 1 in your submission contain map and satellite images which may be copyrighted. All PLOS content is published under the Creative Commons Attribution License (CC BY 4.0), which means that the manuscript, images, and Supporting Information files will be freely available online, and any third party is permitted to access, download, copy, distribute, and use these materials in any way, even commercially, with proper attribution. For these reasons, we cannot publish previously copyrighted maps or satellite images created using proprietary data, such as Google software (Google Maps, Street View, and Earth). For more information, see our copyright guidelines: http://journals.plos.org/plosone/s/licenses-and-copyright.

We require you to either present written permission from the copyright holder to publish these figures specifically under the CC BY 4.0 license, or remove the figures from your submission:

a. You may seek permission from the original copyright holder of Figure 1 to publish the content specifically under the CC BY 4.0 license.

We recommend that you contact the original copyright holder with the Content Permission Form (http://journals.plos.org/plosone/s/file?id=7c09/content-permission-form.pdf) and the following text:

“I request permission for the open-access journal PLOS ONE to publish XXX under the Creative Commons Attribution License (CCAL) CC BY 4.0 (http://creativecommons.org/licenses/by/4.0/). Please be aware that this license allows unrestricted use and distribution, even commercially, by third parties. Please reply and provide explicit written permission to publish XXX under a CC BY license and complete the attached form.”

Please upload the completed Content Permission Form or other proof of granted permissions as an "Other" file with your submission.

In the figure caption of the copyrighted figure, please include the following text: “Reprinted from [ref] under a CC BY license, with permission from [name of publisher], original copyright [original copyright year].”

b. If you are unable to obtain permission from the original copyright holder to publish these figures under the CC BY 4.0 license or if the copyright holder’s requirements are incompatible with the CC BY 4.0 license, please either i) remove the figure or ii) supply a replacement figure that complies with the CC BY 4.0 license. Please check copyright information on all replacement figures and update the figure caption with source information. If applicable, please specify in the figure caption text when a figure is similar but not identical to the original image and is therefore for illustrative purposes only.

The following resources for replacing copyrighted map figures may be helpful:

USGS National Map Viewer (public domain): http://viewer.nationalmap.gov/viewer/

The Gateway to Astronaut Photography of Earth (public domain): http://eol.jsc.nasa.gov/sseop/clickmap/

Maps at the CIA (public domain): https://www.cia.gov/library/publications/the-world-factbook/index.html and https://www.cia.gov/library/publications/cia-maps-publications/index.html

NASA Earth Observatory (public domain): http://earthobservatory.nasa.gov/

Landsat: http://landsat.visibleearth.nasa.gov/

USGS EROS (Earth Resources Observatory and Science (EROS) Center) (public domain): http://eros.usgs.gov/#

Natural Earth (public domain): http://www.naturalearthdata.com/

9. We note that Figures 2, 3 and 4 in your submission contain copyrighted images. All PLOS content is published under the Creative Commons Attribution License (CC BY 4.0), which means that the manuscript, images, and Supporting Information files will be freely available online, and any third party is permitted to access, download, copy, distribute, and use these materials in any way, even commercially, with proper attribution. For more information, see our copyright guidelines: http://journals.plos.org/plosone/s/licenses-and-copyright.

We require you to either present written permission from the copyright holder to publish these figures specifically under the CC BY 4.0 license, or remove the figures from your submission:

a. You may seek permission from the original copyright holder of Figures 2, 3 and 4 to publish the content specifically under the CC BY 4.0 license.

We recommend that you contact the original copyright holder with the Content Permission Form (http://journals.plos.org/plosone/s/file?id=7c09/content-permission-form.pdf) and the following text:

“I request permission for the open-access journal PLOS ONE to publish XXX under the Creative Commons Attribution License (CCAL) CC BY 4.0 (http://creativecommons.org/licenses/by/4.0/). Please be aware that this license allows unrestricted use and distribution, even commercially, by third parties. Please reply and provide explicit written permission to publish XXX under a CC BY license and complete the attached form.”

Please upload the completed Content Permission Form or other proof of granted permissions as an "Other" file with your submission.

In the figure caption of the copyrighted figure, please include the following text: “Reprinted from [ref] under a CC BY license, with permission from [name of publisher], original copyright [original copyright year].”

b. If you are unable to obtain permission from the original copyright holder to publish these figures under the CC BY 4.0 license or if the copyright holder’s requirements are incompatible with the CC BY 4.0 license, please either i) remove the figure or ii) supply a replacement figure that complies with the CC BY 4.0 license. Please check copyright information on all replacement figures and update the figure caption with source information. If applicable, please specify in the figure caption text when a figure is similar but not identical to the original image and is therefore for illustrative purposes only.

10. Please include captions for your Supporting Information files at the end of your manuscript, and update any in-text citations to match accordingly. Please see our Supporting Information guidelines for more information: http://journals.plos.org/plosone/s/supporting-information.

11. If the reviewer comments include a recommendation to cite specific previously published works, please review and evaluate these publications to determine whether they are relevant and should be cited. There is no requirement to cite these works unless the editor has indicated otherwise.

**Additional Editor Comments:**

Dear Dr. Mermelstein,

After the reviews made by independent reviewers, below I indicate the necessary changes you and your co-authors need to do to deliver a new version of your manuscript.

The reviewers recognize this manuscript as a valuable and potentially publishable contribution. The six-year inventory documents substantial biological diversity in a highly threatened Atlantic Forest landscape and demonstrates the usefulness of photography, acoustic recordings, camera traps, digital platforms, and AI-assisted tools for biodiversity monitoring. The resulting dataset has clear potential for conservation, environmental education, and future research. However, substantial revisions are required before the manuscript can be considered for publication.

The principal issue concerns the study’s characterization as “citizen science.” The manuscript must clearly explain who collected the observations, how other participants contributed, and how the iNaturalist community supported taxonomic identification and validation. The authors should quantify this contribution where possible, including the number of identifiers involved, the proportion of observations reaching Research Grade, and examples of records improved through community review. The manuscript should also explain that eligible Research Grade records are incorporated into databases such as GBIF and SIBBr.

A clearer distinction is also needed between AI-generated suggestions and identifications validated by researchers, specialists, or the iNaturalist community. The workflow involving iNaturalist, Merlin, Addax AI, and human verification should be described transparently, together with its limitations. Claims about AI-based citizen science should then be revised to accurately reflect the methods used.

The taxonomic scope also requires reconsideration. Analyses of native, exotic, endemic, threatened, or invasive status should preferably be restricted to organisms identified at the species level, because genus-level identifications may not permit reliable classification. The authors should clearly report the taxonomic resolution and validation status of the records included in each analysis.

Finally, the ecological and geographical context should be strengthened by describing nearby protected areas and explaining how the study site may contribute to a regional biodiversity corridor. Evidence such as the puma and other wide-ranging mammals may support this interpretation, but conclusions about environmental health, connectivity, or conservation value should remain appropriately cautious.

Given the importance of these conceptual and methodological clarifications—particularly because citizen science and AI are central to the title, abstract, and discussion—I recommend Major Revision. A revised manuscript that transparently defines the data-collection and identification workflow, adjusts unsupported claims, and strengthens the regional conservation context could represent a useful contribution to biodiversity documentation in the Atlantic Forest.

Sincerely,

Daniel Silva

Reviewers' comments:

**Comments to the Author**

1. Is the manuscript technically sound, and do the data support the conclusions?

Reviewer #1: Yes

Reviewer #2: Yes

**********

2. Has the statistical analysis been performed appropriately and rigorously?

Reviewer #1: N/A

Reviewer #2: Yes

**********

3. Have the authors made all data underlying the findings in their manuscript fully available?

The PLOS Data policy

Reviewer #1: Yes

Reviewer #2: Yes

**********

4. Is the manuscript presented in an intelligible fashion and written in standard English?

Reviewer #1: No

Reviewer #2: Yes

**********

**Reviewer #1:**  This manuscript presents a valuable biodiversity inventory of an Atlantic Forest region in Brazil based on six years of observations using photography, acoustic recordings, camera traps, and AI-assisted identification tools. The study documents a remarkable diversity of organisms and demonstrates the practical potential of modern digital technologies for biodiversity monitoring. The resulting dataset will likely be useful for conservation, environmental education, and future ecological studies.

The manuscript is generally well written and the long-term commitment required to generate this dataset deserves recognition. Nevertheless, I believe that several important conceptual issues should be clarified before the manuscript can be considered for publication.

The central concern relates to the use of the term “citizen science.” Throughout the manuscript, citizen science is presented as one of the major foundations of the study. However, the methods section does not clearly describe the participation of citizen scientists in the data collection process. Based on the current text, it appears that most observations were collected directly by the authors and subsequently uploaded to platforms such as iNaturalist. Although these platforms are important citizen-science infrastructures, the use of a citizen-science platform is not necessarily equivalent to conducting a citizen-science study. Therefore, I encourage the authors to explain more explicitly how citizen science contributed to this work. Clarifying who collected the observations, whether additional participants contributed records, and how community members participated in species validation would strengthen the manuscript considerably.

Related to this issue, the manuscript would benefit from a clearer description of the role played by the iNaturalist community. The study mentions that numerous observations were reviewed and validated by other users. This is potentially one of the strongest citizen-science components of the work. The authors may wish to provide quantitative information regarding community participation, such as the number of identifiers involved, the proportion of records reaching Research Grade status, or examples in which community review improved species identification. Such information would provide direct evidence of citizen-science contributions rather than relying primarily on a conceptual discussion.

A second issue concerns the role of artificial intelligence. The manuscript appropriately highlights the use of iNaturalist, Merlin, and Addax AI as important tools. However, the distinction between AI-generated identifications and human validation is not always clear. In practice, many biodiversity observations on citizen-science platforms are produced through an interaction between automated suggestions and expert review. I encourage the authors to clarify this workflow and to discuss the respective contributions of AI and human expertise. This clarification would help readers better understand the strengths and limitations of the approach.

Finally, some statements throughout the manuscript appear stronger than what is directly supported by the methodology. In particular, claims regarding AI-based citizen science should be phrased carefully unless the citizen-science component and the contribution of AI can be more explicitly demonstrated. I encourage the authors to revise these statements where appropriate and ensure that the terminology accurately reflects the study design.

Overall, I consider this work to be a valuable contribution and potentially suitable for publication after revision. Clarifying the citizen-science framework and the role of AI would substantially strengthen the manuscript and improve its scientific transparency.

**Reviewer #2:**  This is an unprecedented work based on records of animals, plants, and fungi collected over 6 years on a private site in the rural area of Serra do Mar, which still holds remnants of the Atlantic Forest, the most impacted biome in Brazil. Nearby, there are established conservation units, and in the rural areas, biodiversity corridors connect these areas.

The species identification strategy on the iNaturalist platform gives it a citizen science character, with interaction between the record authors and specialists for each group of organisms.

Thus, in a collaborative way, many records were able to identify the organism at the species level.

To reach the Research Grade (RG), at least 2 different iNaturalists must agree on the identification and there must be no conflicting identifications. All records that achieve RG will automatically be made available by iNaturalist in the GBIF global database, with each 'observation' available 'individually' for various research purposes.

Having hundreds of organism records in an area is relevant, but it doesn't allow us to assess the degree of biodiversity preservation or any special characteristics. That was exactly the aim of this work, which makes biodiversity data available to environmental agencies and for environmental education activities.

For the region where the study is located, this is a unique effort, and at the Atlantic Forest level, it represents an innovative approach, especially with the combination of records from cameras, cell phones, and particularly fixed cameras.

The compilation of records indicates that the portion of the Atlantic Forest on the private site still maintains a high level of native biodiversity. Reviewing the data down to the species level, we found that 88% of the records are of native Atlantic Forest organisms, with 10% being endemic species and 4% threatened species. On the other hand, we observed 12% of records of exotic species, with 5.4% being invasive species.

In a paper recently approved for a conference in August 2026 of the Brazilian Citizen Science Network, considering the 20 Brazilian projects participating in the City Nature Challenge 2026 on iNaturalist, we found that 25% of the 200 most recorded species were exotic species. This pattern was consistent in the number of records and indicates that the human impact on urban areas and native areas around urban spaces is progressing rapidly, and measures are needed to slow this advance.

In particular, the records of the puma, which tends to roam across several square kilometers of forest and wouldn’t survive in the small patch of Atlantic Forest where the site is located, indicate that this is land situated within a "Biodiversity Corridor" connecting other native areas. The wide variety of terrestrial mammals captured by the camera traps also supports this characteristic.

I work at Ibama (National Environmental Agency) and I understand the difficulty of finding areas to release animals that have been seized and rehabilitated at Wild Animal Screening Centers - CETAS. Few studies on selecting release areas have the breadth and detail of the survey conducted. The data is qualitative, but it already gives an indication of 'environmental health,' and some quantitative studies are needed to determine which species could be released. It's just an example of the positive potential of this type of survey using citizen science tools.

Here are some Recommendations:

1) The work should focus on organisms identified at the species level, since organisms identified only at the genus level don’t allow for accuracy in determining whether they are native, exotic, endemic, or invasive species.

2) The work should include information about nearby conservation units and the integration of the private site into a biodiversity corridor.

3) Include in the text the strategy for identifying species recorded on iNaturalist through citizen science, and highlight the exceptionality of Research Grade records being made available in the global GBIF databases and the national SIBBr database.

**********

what does this mean?). If published, this will include your full peer review and any attached files.

If you choose “no”, your identity will remain anonymous but your review may still be made public.

**Do you want your identity to be public for this peer review?** For information about this choice, including consent withdrawal, please see our Privacy Policy

Reviewer #1: **Yes:**  Vincent Y. Chen

Reviewer #2: **Yes:**  Eric Fischer Rempe

**********

[NOTE: If reviewer comments were submitted as an attachment file, they will be attached to this email and accessible via the submission site. Please log into your account, locate the manuscript record, and check for the action link "View Attachments". If this link does not appear, there are no attachment files.]

To ensure your figures meet our technical requirements, please review our figure guidelines: https://journals.plos.org/plosone/s/figures

You may also use PLOS’s free figure tool, NAAS, to help you prepare publication quality figures: https://journals.plos.org/plosone/s/figures#loc-tools-for-figure-preparation.

NAAS will assess whether your figures meet our technical requirements by comparing each figure against our figure specifications.

# Author comment: Author response to Decision Letter 1

**DOI:** 10.1371/journal.pone.0360070.r002

23 Aug 2026

Dear Dr. Daniel de Paiva Silva,

We submitted the manuscript "Integrating images, sounds, citizen science and AI to assess the biodiversity of the Atlantic Forest of Brazil” (PONE-D-26-15035) by Costa et al., for publication in Plos One. The reviewers recommended major revisions to the manuscript. We thank the Reviewers for their careful and appropriate analysis. We included a point-by-point response to the reviewer’s comments, which were useful to improve the data presentation and interpretation. The detailed corrections are as follows.

Journal Requirements:

1. When submitting your revision, we need you to address these additional requirements.

Please ensure that your manuscript meets PLOS ONE's style requirements, including those for file naming. The PLOS ONE style templates can be found at

https://journals.plos.org/plosone/s/file?id=wjVg/PLOSOne_formatting_sample_main_body.pdf and

https://journals.plos.org/plosone/s/file?id=ba62/PLOSOne_formatting_sample_title_authors_affiliations.pdf

Author’s response: We thank the editor for this comment. We apologize for submitting with the wrong format, and we adjusted the style accordingly.

2. In your Methods section, please provide additional information regarding the permits you obtained for the work. Please ensure you have included the full name of the authority that approved the field site access and, if no permits were required, a brief statement explaining why.

Author’s response: We thank the reviewer for this important remark. In fact, we own the property, and therefore no authorization was needed. We changed the text to make this information clear.

3. Please note that PLOS One has specific guidelines on code sharing for submissions in which author-generated code underpins the findings in the manuscript. In these cases, we expect all author-generated code to be made available without restrictions upon publication of the work. Please review our guidelines at https://journals.plos.org/plosone/s/materials-and-software-sharing#loc-sharing-code and ensure that your code is shared in a way that follows best practice and facilitates reproducibility and reuse.

Author’s response: We did not generate any code; we only used the mentioned software.

4. We note that the grant information you provided in the ‘Funding Information’ and ‘Financial Disclosure’ sections do not match.

When you resubmit, please ensure that you provide the correct grant numbers for the awards you received for your study in the ‘Funding Information’ section.

Author’s response: We corrected this.

5. Thank you for stating in your Funding Statement:

This work was supported by grants from Conselho Nacional de Desenvolvimento Científico e Tecnológico (CNPq, funding number 302961/2021-6 to C.M., 308192/2021-4 to M.L.C.) and Fundação de Apoio à Pesquisa do Estado do Rio de Janeiro (FAPERJ, funding number E-26/203.930/2024 to C.M., E26/204.077/2024 to M.L.C.).

Please provide an amended statement that declares *all* the funding or sources of support (whether external or internal to your organization) received during this study, as detailed online in our guide for authors at http://journals.plos.org/plosone/s/submit-now. Please also include the statement “There was no additional external funding received for this study.” in your updated Funding Statement.

Please include your amended Funding Statement within your cover letter. We will change the online submission form on your behalf.

Author’s response: We thank the editor for this remark, and we corrected the text accordingly.

6. Thank you for stating the following financial disclosure:

This work was supported by grants from Conselho Nacional de Desenvolvimento Científico e Tecnológico (CNPq, funding number 302961/2021-6 to C.M., 308192/2021-4 to M.L.C.) and Fundação de Apoio à Pesquisa do Estado do Rio de Janeiro (FAPERJ, funding number E-26/203.930/2024 to C.M., E26/204.077/2024 to M.L.C.).

Please state what role the funders took in the study. If the funders had no role, please state: "The funders had no role in study design, data collection and analysis, decision to publish, or preparation of the manuscript."

If this statement is not correct you must amend it as needed.

Please include this amended Role of Funder statement in your cover letter; we will change the online submission form on your behalf.

Author’s response: We thank the editor for this remark, and we corrected the text accordingly.

7. Thank you for uploading your study's underlying data set. Unfortunately, the repository you have noted in your Data Availability statement does not qualify as an acceptable data repository according to PLOS's standards.

At this time, please upload the minimal data set necessary to replicate your study's findings to a stable, public repository (such as figshare or Dryad) and provide us with the relevant URLs, DOIs, or accession numbers that may be used to access these data. For a list of recommended repositories and additional information on PLOS standards for data deposition, please see https://journals.plos.org/plosone/s/recommended-repositories.

Author’s response: We thank the editor for this remark. We corrected the manuscript to explain that all data is available in the supplementary file.

8. We note that Figure 1 in your submission contains map and satellite images which may be copyrighted. All PLOS content is published under the Creative Commons Attribution License (CC BY 4.0), which means that the manuscript, images, and Supporting Information files will be freely available online, and any third party is permitted to access, download, copy, distribute, and use these materials in any way, even commercially, with proper attribution. For these reasons, we cannot publish previously copyrighted maps or satellite images created using proprietary data, such as Google software (Google Maps, Street View, and Earth). For more information, see our copyright guidelines: http://journals.plos.org/plosone/s/licenses-and-copyright.

We require you to either present written permission from the copyright holder to publish these figures specifically under the CC BY 4.0 license, or remove the figures from your submission:

a. You may seek permission from the original copyright holder of Figure 1 to publish the content specifically under the CC BY 4.0 license.

We recommend that you contact the original copyright holder with the Content Permission Form (http://journals.plos.org/plosone/s/file?id=7c09/content-permission-form.pdf) and the following text:

“I request permission for the open-access journal PLOS ONE to publish XXX under the Creative Commons Attribution License (CCAL) CC BY 4.0 (http://creativecommons.org/licenses/by/4.0/). Please be aware that this license allows unrestricted use and distribution, even commercially, by third parties. Please reply and provide explicit written permission to publish XXX under a CC BY license and complete the attached form.”

Please upload the completed Content Permission Form or other proof of granted permissions as an "Other" file with your submission.

In the figure caption of the copyrighted figure, please include the following text: “Reprinted from [ref] under a CC BY license, with permission from [name of publisher], original copyright [original copyright year].”

b. If you are unable to obtain permission from the original copyright holder to publish these figures under the CC BY 4.0 license or if the copyright holder’s requirements are incompatible with the CC BY 4.0 license, please either i) remove the figure or ii) supply a replacement figure that complies with the CC BY 4.0 license. Please check copyright information on all replacement figures and update the figure caption with source information. If applicable, please specify in the figure caption text when a figure is similar but not identical to the original image and is therefore for illustrative purposes only.

The following resources for replacing copyrighted map figures may be helpful:

USGS National Map Viewer (public domain): http://viewer.nationalmap.gov/viewer/

The Gateway to Astronaut Photography of Earth (public domain): http://eol.jsc.nasa.gov/sseop/clickmap/

Maps at the CIA (public domain): https://www.cia.gov/library/publications/the-world-factbook/index.html and https://www.cia.gov/library/publications/cia-maps-publications/index.html

NASA Earth Observatory (public domain): http://earthobservatory.nasa.gov/

Landsat: http://landsat.visibleearth.nasa.gov/

USGS EROS (Earth Resources Observatory and Science (EROS) Center) (public domain): http://eros.usgs.gov/#

Natural Earth (public domain): http://www.naturalearthdata.com/

Author’s response: We thank the editor for this remark, and we prepared a new Figure 1 and a new figure legend. The image of Brazil in Figure 1A was downloaded from a public domain site. Figure 1B was obtained from Instituto Brasileiro de Geografia e Estatística, IBGE, which has an open data policy. Figure 1C was downloaded from Sentinel 2 L2a at Copernicus Sentinel Data and Service Information, which has an open data policy. We added information about the sources in the figure legend.

9. We note that Figures 2, 3 and 4 in your submission contain copyrighted images. All PLOS content is published under the Creative Commons Attribution License (CC BY 4.0), which means that the manuscript, images, and Supporting Information files will be freely available online, and any third party is permitted to access, download, copy, distribute, and use these materials in any way, even commercially, with proper attribution. For more information, see our copyright guidelines: http://journals.plos.org/plosone/s/licenses-and-copyright.

We require you to either present written permission from the copyright holder to publish these figures specifically under the CC BY 4.0 license, or remove the figures from your submission:

a. You may seek permission from the original copyright holder of Figures 2, 3 and 4 to publish the content specifically under the CC BY 4.0 license.

We recommend that you contact the original copyright holder with the Content Permission Form (http://journals.plos.org/plosone/s/file?id=7c09/content-permission-form.pdf) and the following text:

“I request permission for the open-access journal PLOS ONE to publish XXX under the Creative Commons Attribution License (CCAL) CC BY 4.0 (http://creativecommons.org/licenses/by/4.0/). Please be aware that this license allows unrestricted use and distribution, even commercially, by third parties. Please reply and provide explicit written permission to publish XXX under a CC BY license and complete the attached form.”

Please upload the completed Content Permission Form or other proof of granted permissions as an "Other" file with your submission.

In the figure caption of the copyrighted figure, please include the following text: “Reprinted from [ref] under a CC BY license, with permission from [name of publisher], original copyright [original copyright year].”

b. If you are unable to obtain permission from the original copyright holder to publish these figures under the CC BY 4.0 license or if the copyright holder’s requirements are incompatible with the CC BY 4.0 license, please either i) remove the figure or ii) supply a replacement figure that complies with the CC BY 4.0 license. Please check copyright information on all replacement figures and update the figure caption with source information. If applicable, please specify in the figure caption text when a figure is similar but not identical to the original image and is therefore for illustrative purposes only.

Author’s response: We thank the editor for this remark. We corrected the copyright requirements of all the images in the iNaturalist platform that we used in the manuscript to comply with the CC BY 4.0 license.

10. Please include captions for your Supporting Information files at the end of your manuscript, and update any in-text citations to match accordingly. Please see our Supporting Information guidelines for more information: http://journals.plos.org/plosone/s/supporting-information.

Author’s response: We thank the editor for this remark, and we apologize for this mistake. We provided a proper caption for the Supplementary File.

11. If the reviewer’s comments include a recommendation to cite specific previously published works, please review and evaluate these publications to determine whether they are relevant and should be cited. There is no requirement to cite these works unless the editor has indicated otherwise.

Author’s response: The reviewers did not recommend to cite specific previously published works.

Additional Editor Comments:

Dear Dr. Mermelstein,

After the reviews made by independent reviewers, below I indicate the necessary changes you and your co-authors need to do to deliver a new version of your manuscript.

The reviewers recognize this manuscript as a valuable and potentially publishable contribution. The six-year inventory documents substantial biological diversity in a highly threatened Atlantic Forest landscape and demonstrates the usefulness of photography, acoustic recordings, camera traps, digital platforms, and AI-assisted tools for biodiversity monitoring. The resulting dataset has clear potential for conservation, environmental education, and future research. However, substantial revisions are required before the manuscript can be considered for publication.

12. The principal issue concerns the study’s characterization as “citizen science.” The manuscript must clearly explain who collected the observations, how other participants contributed, and how the iNaturalist community supported taxonomic identification and validation.

Author’s response: We thank the editor for this comment, which we agree should be clarified. All the observations and data organization were conducted by the authors solely, and no other participants contributed. We included this information in the manuscript. The whole iNat community contributed to the study, through their participation in the identification of species. We incorporated the discussion about CS in the manuscript. We assume the study qualify as CS because: 1) We propose that anyone with access to a private property can conduct a similar study; 2) The study was conceived and conducted by scientists that have been trained in a different area; and 3) Communities of citizens such as Merlin and iNat participated in the classification.

13. The authors should quantify this contribution where possible, including the number of identifiers involved, the proportion of observations reaching Research Grade, and examples of records improved through community review.

Author’s response: We thank the editor for these comments. We now include the number of identifiers involved (495) and the proportion of observations reaching Research Grade in the first paragraph of the Results section. We also added an example of a record improved through the iNat community in the “mammals” section of the Results.

14. The manuscript should also explain that eligible Research Grade records are incorporated into databases such as GBIF and SIBBr.

Author’s response: We thank the editor for this comment. We incorporated this discussion in the manuscript. In fact, 2000 of our observations were incorporated into the GBIF database. Furthermore, we register on the SIBBr database.

15. A clearer distinction is also needed between AI-generated suggestions and identifications validated by researchers, specialists, or the iNaturalist community. The workflow involving iNaturalist, Merlin, Addax AI, and human verification should be described transparently, together with its limitations. Claims about AI-based citizen science should then be revised to accurately reflect the methods used.

Author’s response: We thank the editor for this comment. We agree that the workflow should be clarified. We included this description in the Material and Methods section, and we introduced a new figure to depict it.

16. The taxonomic scope also requires reconsideration. Analyses of native, exotic, endemic, threatened, or invasive status should preferably be restricted to organisms identified at the species level, because genus-level identifications may not permit reliable classification. The authors should clearly report on the taxonomic resolution and validation status of the records included in each analysis.

Author’s response: We thank the editor for this comment. We agree that the analyses of status should be restricted to organisms identified at the species level. That is why in our manuscript we only refer to the introduced or threatened status of organisms identified at the species level, including in Table 6 (related to plant species) and Table 11 (related to threatened species).

17. Finally, the ecological and geographical context should be strengthened by describing nearby protected areas and explaining how the study site may contribute to a regional biodiversity corridor. Evidence such as the puma and other wide-ranging mammals may support this interpretation, but conclusions about environmental health, connectivity, or conservation value should remain appropriately cautious.

Author’s response: We thank the editor for this comment. We were already mentioning the proximity to protected areas in the description of the place in the methods section. Now we have added a discussion of how the puma could be moving between these areas.

Given the importance of these conceptual and methodological clarifications—particularly because citizen science and AI are central to the title, abstract, and discussion, I recommend Major Revision. A revised manuscript that transparently defines the data-collection and identification workflow, adjusts unsupported claims, and strengthens the regional conservation context, could represent a useful contribution to biodiversity documentation in the Atlantic Forest.

We hope that the modifications made in the new version of the manuscript have properly addressed the criticism and suggestions made by the reviewer, and that the improvements made in the manuscript will be sufficient for its publication in Plos One.

We would like to state that all listed authors qualify for authorship and agree with the submission of the revised version of the manuscript. The final version of the manuscript has been seen and approved by all coauthors. The authors declare that they have no conflict of interest.

With kind regards,

Claudia Mermelstein

Attachment Submitted filename: Response to Reviewers.docx

# Peer review: Decision Letter 1

**DOI:** 10.1371/journal.pone.0360070.r003

**Authors:** Daniel Silva

31 Aug 2026

Dear Dr. Mermelstein,

Thank you for submitting your manuscript to PLOS One. After careful consideration, we feel that it has merit but does not fully meet PLOS One’s publication criteria as it currently stands. Therefore, we invite you to submit a revised version of the manuscript that addresses the points raised during the review process.

Please submit your revised manuscript by Oct 15 2026 11:59PM. If you will need more time than this to complete your revisions, please reply to this message or contact the journal office at plosone@plos.org. When you're ready to submit your revision, log on to https://www.editorialmanager.com/pone/ and select the 'Submissions Needing Revision' folder to locate your manuscript file.

- A letter that responds to each point raised by the academic editor and reviewer(s). You should upload this letter as a separate file labeled 'Response to Reviewers'.
- A marked-up copy of your manuscript that highlights changes made to the original version. You should upload this as a separate file labeled 'Revised Manuscript with Track Changes'.
- An unmarked version of your revised paper without tracked changes. You should upload this as a separate file labeled 'Manuscript'.

If you would like to make changes to your financial disclosure, please include your updated statement in your cover letter. Guidelines for resubmitting your figure files are available below the reviewer comments at the end of this letter.

If applicable, we recommend that you deposit your laboratory protocols in protocols.io to enhance the reproducibility of your results. Protocols.io assigns your protocol its own identifier (DOI) so that it can be cited independently in the future. For instructions see: https://journals.plos.org/plosone/s/submission-guidelines#loc-laboratory-protocols. Additionally, PLOS One offers an option for publishing peer-reviewed Lab Protocol articles, which describe protocols hosted on protocols.io. Read more information on sharing protocols at https://plos.org/protocols?utm_medium=editorial-email&utm_source=authorletters&utm_campaign=protocols.

As the corresponding author, your ORCID iD is verified in the submission system and will appear in the published article. PLOS supports the use of ORCID, and we encourage all coauthors to register for an ORCID iD and use it as well. Please encourage your coauthors to verify their ORCID iD within the submission system before final acceptance, as unverified ORCID iDs will not appear in the published article. *Only* the individual author can complete the verification step; PLOS staff *cannot* verify ORCID iDs on behalf of authors.

We look forward to receiving your revised manuscript.

Kind regards,

Daniel de Paiva Silva, Ph.D.

Academic Editor

PLOS One

Journal Requirements:

If the reviewer comments include a recommendation to cite specific previously published works, please review and evaluate these publications to determine whether they are relevant and should be cited. There is no requirement to cite these works unless the editor has indicated otherwise.

Please review your reference list to ensure that it is complete and correct. If you have cited papers that have been retracted, please include the rationale for doing so in the manuscript text, or remove these references and replace them with relevant current references. Any changes to the reference list should be mentioned in the rebuttal letter that accompanies your revised manuscript. If you need to cite a retracted article, indicate the article’s retracted status in the References list and also include a citation and full reference for the retraction notice.

Additional Editor Comments :

Dear Dr. Mermelstein,

After a new review round of our manuscript, both reviewers indicated that substantial changes were made and that your manuscript is worth of publication in PLoS One. Still, Reviewer #1 (for once, it was not Reviewer #2 at this time) required some minor changes to be made in a new version. As soon as the changes are made, I believe your manuscript will be formally accepted for publication in PLoS One.

Sincerely,

Daniel SIlva

[Note: HTML markup is below. Please do not edit.]

Reviewers' comments:

Reviewer's Responses to Questions

**Comments to the Author**

Reviewer #1: All comments have been addressed

Reviewer #2: All comments have been addressed

**********

2. Is the manuscript technically sound, and do the data support the conclusions?

Reviewer #1: Yes

Reviewer #2: Yes

**********

3. Has the statistical analysis been performed appropriately and rigorously?

Reviewer #1: N/A

Reviewer #2: N/A

**********

4. Have the authors made all data underlying the findings in their manuscript fully available?

The PLOS Data policy

Reviewer #1: Yes

Reviewer #2: Yes

**********

5. Is the manuscript presented in an intelligible fashion and written in standard English?

Reviewer #1: Yes

Reviewer #2: Yes

**********

Reviewer #1: Thank you for the careful and substantial revision of the manuscript. The revised version has addressed most of my previous concerns, particularly those regarding the definition of the citizen-science component and the respective roles of artificial intelligence and human validation.

I appreciate the clarification that all images, sounds, and primary observations were collected by the two authors, while the broader citizen-science contribution occurred primarily through the iNaturalist community and related identification platforms. The inclusion of 495 iNaturalist identifiers, together with the taxon-specific proportions of observations reaching Research Grade, provides a much clearer and more quantitative description of community participation. This is a significant improvement over the previous version.

The revised description of the identification workflow is also much stronger. The manuscript now makes it clear that automated tools such as iNaturalist, Merlin, and Addax AI assist with identification or data processing, while final classification may involve researchers, experienced naturalists, specialists, and subsequent manual verification. The example involving Callithrix hybrids is particularly informative because it illustrates how community-based expertise can refine or surpass automated AI classification.

The discussion of taxonomic resolution and the differences in Research Grade percentages among organism groups is also helpful, as is the expanded consideration of nearby protected areas and the possible regional conservation significance of the study site. These revisions improve both the methodological transparency and the ecological context of the manuscript.

I have only two remaining minor suggestions.

First, I recommend slightly moderating the use of the phrase “AI-based citizen science approach,” particularly in the Abstract and other prominent sections. Since the field observations were generated solely by the authors, while citizen participation occurred mainly during identification and validation through platforms such as iNaturalist, the current wording may still imply a broader citizen-science data-collection effort than was actually conducted. A more precise formulation might be “an integrated approach combining digital observations, AI-assisted species identification, and citizen-science community validation.” This wording would, in my view, better reflect the actual strengths of the study.

Second, please clarify the apparent difference between the 1,515 iNaturalist records downloaded for analysis and the statement that approximately 2,000 observations were incorporated into GBIF. If these numbers refer to different time points, subsets, or database synchronization processes, a brief explanation would be sufficient.

Overall, the manuscript has been substantially improved, and the authors have responded appropriately to the major concerns raised in the previous review. I therefore recommend Minor Revision.

Reviewer #2: All my technical recommendations were considered.

The high biodiversity of native species found indicates a high degree of preservation of the ecosystems existing on the property, resulting from the preservation-oriented land-use practices maintained by the authors' family since 1960. Conversely, the presence of introduced species—some of which are invasive—indicates growing anthropogenic pressure on the area. Thus, this study demonstrates that private properties can contribute significantly to the preservation of Atlantic Forest biodiversity, while also highlighting the need to adopt measures to combat invasive species.

Although this work was conducted on a citizen science platform and the authors are neither zoologists nor botanists, the accuracy of species identification is extremely high—owing both to the authors' own precision and to the confirmation provided by "top identifiers" (mostly specialists) in the most complex or difficult cases. A large proportion of the species are quite common and easy to identify—such as the records of pumas.

As a frequent user of the iNaturalist platform, I can attest that various records made on the private property—for which a species identification had not yet been reached—were not included by the authors. All records from the six-year period, as well as more recent records, can be freely accessed on iNaturalist.

So, I recommend that this work, once published, be forwarded to the National Research and Conservation Center for Carnivorous Mammals (Cenap/ICMBio)—which develops the National Action Plan for the Conservation of Large Cats (PAN Grandes Felinos)—reporting the dates and times of the recorded *Puma* sightings.

**********

what does this mean?). If published, this will include your full peer review and any attached files.

If you choose “no”, your identity will remain anonymous but your review may still be made public.

**Do you want your identity to be public for this peer review?** For information about this choice, including consent withdrawal, please see our Privacy Policy

Reviewer #1: **Yes:**  Vincent Y. Chen

Reviewer #2: **Yes:**  Eric Fischer Rempe

**********

[NOTE: If reviewer comments were submitted as an attachment file, they will be attached to this email and accessible via the submission site. Please log into your account, locate the manuscript record, and check for the action link "View Attachments". If this link does not appear, there are no attachment files.]

To ensure your figures meet our technical requirements, please review our figure guidelines: https://journals.plos.org/plosone/s/figures

You may also use PLOS’s free figure tool, NAAS, to help you prepare publication quality figures: https://journals.plos.org/plosone/s/figures#loc-tools-for-figure-preparation.

NAAS will assess whether your figures meet our technical requirements by comparing each figure against our figure specifications.

# Author comment: Author response to Decision Letter 2

**DOI:** 10.1371/journal.pone.0360070.r004

1 Sep 2026

Rio de Janeiro, September 01, 2026.

Dear Dr. Daniel de Paiva Silva,

We submitted the manuscript "Integrating images, sounds, citizen science and AI to assess the biodiversity of the Atlantic Forest of Brazil” (PONE-D-26-15035R1) by Costa et al., for publication in Plos One. The reviewers recommended minor revisions to the manuscript. We thank the Reviewers for their careful and appropriate analysis. We included a point-by-point response to the reviewer’s comments, which were useful for improving the data presentation and interpretation. The detailed corrections are as follows.

Journal Requirements:

1 - If the reviewer’s comments include a recommendation to cite specific previously published works, please review and evaluate these publications to determine whether they are relevant and should be cited. There is no requirement to cite these works unless the editor has indicated otherwise.

Author’s response: We did not find any recommendations from the reviewers to cite specific previously published works.

2 - Please review your reference list to ensure that it is complete and correct. If you have cited papers that have been retracted, please include the rationale for doing so in the manuscript text, or remove these references and replace them with relevant current references. Any changes to the reference list should be mentioned in the rebuttal letter that accompanies your revised manuscript. If you need to cite a retracted article, indicate the article’s retracted status in the References list and also include a citation and full reference for the retraction notice.

Author’s response: We reviewed our reference list, and it is complete and correct. We are not citing retracted papers. We confirmed that by checking all DOI numbers from the cited papers in the software Retraction Watch Database (https://retractiondatabase.org/RetractionSearch.aspx).

Reviewer #1:

1 - First, I recommend slightly moderating the use of the phrase “AI-based citizen science approach,” particularly in the Abstract and other prominent sections. Since the field observations were generated solely by the authors, while citizen participation occurred mainly during identification and validation through platforms such as iNaturalist, the current wording may still imply a broader citizen-science data-collection effort than was actually conducted. A more precise formulation might be “an integrated approach combining digital observations, AI-assisted species identification, and citizen-science community validation.” This wording would, in my view, better reflect the actual strengths of the study.

Author’s response: We thank the reviewer for this comment. We agree that our paper shows a less conventional approach to Citizen Science. We changed the text in the Abstract according to the reviewer’s suggestions.

2 - Second, please clarify the apparent difference between the 1,515 iNaturalist records downloaded for analysis and the statement that approximately 2,000 observations were incorporated into GBIF. If these numbers refer to different time points, subsets, or database synchronization processes, a brief explanation would be sufficient.

Author’s response: We thank the reviewer for this remark. In fact, we were referring to the total number of observations that we registered in iNaturalist that were included in GBif. The actual number of observations in the studied area with iNaturalist that were included in GBif is 950. We corrected the query parameters used in GBif in the text, using the GBif recommended citation format. We included the following new information in the Results section of the manuscript:

Some of our iNaturalist data were incorporated into other databases, such as in Global Biodiversity Information Facility (950 occurrences in “GBIF, GBIF.org (1 September 2026) GBIF Occurrence Download https://doi.org/10.15468/dl.mm8bxs”, available at https://www.gbif.org/occurrence/search?recordedBy=claudia_mermelstein&geometry=POLYGON%28%28-43.01459+-22.2552%2C-43.01838+-22.2581%2C-43.01303+-22.26484%2C-43.00942+-22.26167%2C-43.01459+-22.2552%29%29 ) and Sistema de Informação sobre a Biodiversidade Brasileira (SiBBr, https://sibbr.gov.br/). These Citizen Science platforms increase the availability of our data.

We hope that the modifications made in the new version of the manuscript have properly addressed the criticism and suggestions made by the reviewer, and that the improvements made in the manuscript will be sufficient for its publication in Plos One.

We would like to state that all listed authors qualify for authorship and agree with the submission of the revised version of the manuscript. The final version of the manuscript has been seen and approved by all coauthors. The authors declare that they have no conflict of interest.

With kind regards,

Claudia Mermelstein

Attachment Submitted filename: Response_to_Reviewers_auresp_2.docx

# Peer review: Decision Letter 2

**DOI:** 10.1371/journal.pone.0360070.r005

**Authors:** Daniel Silva

22 Sep 2026

Integrating images, sounds, citizen science and AI to assess the biodiversity of the Atlantic Forest of Brazil

PONE-D-26-15035R2

Dear Dr. Mermelstein,

We’re pleased to inform you that your manuscript has been judged scientifically suitable for publication and will be formally accepted for publication once it meets all outstanding technical requirements.

Within one week, you’ll receive an e-mail detailing the required amendments. When these have been addressed, you’ll receive a formal acceptance letter and your manuscript will be scheduled for publication.

An invoice will be generated when your article is formally accepted. Please note, if your institution has a publishing partnership with PLOS and your article meets the relevant criteria, all or part of your publication costs will be covered. Please make sure your user information is up-to-date by logging into Editorial Manager at Editorial Manager® and clicking the ‘Update My Information' link at the top of the page. For questions related to billing, please contact billing support.

If your institution or institutions have a press office, please notify them about your upcoming paper to help maximize its impact. If they’ll be preparing press materials, please inform our press team as soon as possible -- no later than 48 hours after receiving the formal acceptance. Your manuscript will remain under strict press embargo until 2 pm Eastern Time on the date of publication. For more information, please contact onepress@plos.org.

Kind regards,

Daniel de Paiva Silva, Ph.D.

Academic Editor

PLOS One

Additional Editor Comments (optional):

Reviewers' comments:

Reviewer's Responses to Questions

**Comments to the Author**

Reviewer #2: All comments have been addressed

**********

2. Is the manuscript technically sound, and do the data support the conclusions?

Reviewer #2: Yes

**********

3. Has the statistical analysis been performed appropriately and rigorously?

Reviewer #2: I Don't Know

**********

4. Have the authors made all data underlying the findings in their manuscript fully available?

The PLOS Data policy

Reviewer #2: Yes

**********

5. Is the manuscript presented in an intelligible fashion and written in standard English?

Reviewer #2: Yes

**********

Reviewer #2: There has been significant evolution from the original text to the current version, incorporating the additions and adjustments made.

**********

what does this mean?). If published, this will include your full peer review and any attached files.

If you choose “no”, your identity will remain anonymous but your review may still be made public.

**Do you want your identity to be public for this peer review?** For information about this choice, including consent withdrawal, please see our Privacy Policy

Reviewer #2: **Yes:**  Eric Fischer Rempe

**********

# Editor report: Acceptance letter

**DOI:** 10.1371/journal.pone.0360070.r006

**Authors:** Daniel Silva

PONE-D-26-15035R2

PLOS One

Dear Dr. Mermelstein,

I'm pleased to inform you that your manuscript has been deemed suitable for publication in PLOS One. Congratulations! Your manuscript is now being handed over to our production team.

At this stage, our production department will prepare your paper for publication. This includes ensuring the following:

* All references, tables, and figures are properly cited

* All relevant supporting information is included in the manuscript submission,

* There are no issues that prevent the paper from being properly typeset

You will receive further instructions from the production team, including instructions on how to review your proof when it is ready. Please keep in mind that we are working through a large volume of accepted articles, so please give us a few days to review your paper and let you know the next and final steps.

Lastly, if your institution or institutions have a press office, please let them know about your upcoming paper now to help maximize its impact. If they'll be preparing press materials, please inform our press team within the next 48 hours. Your manuscript will remain under strict press embargo until 2 pm Eastern Time on the date of publication. For more information, please contact onepress@plos.org.

You will receive an invoice from PLOS for your publication fee after your manuscript has reached the completed accept phase. If you receive an email requesting payment before acceptance or for any other service, this may be a phishing scheme. Learn how to identify phishing emails and protect your accounts at https://explore.plos.org/phishing.

If we can help with anything else, please email us at customercare@plos.org.

Thank you for submitting your work to PLOS One and supporting open access.

Kind regards,

PLOS One Editorial Office Staff

on behalf of

Dr. Daniel de Paiva Silva

Academic Editor

PLOS One
